# Competition between hydrogen atom transfer and carbon radical addition pathways in ClO_2_ oxidation of dihydroxybenzenes for water treatment: a DFT study

**DOI:** 10.1039/d6ra08249a

**Published:** 2026-10-26

**Authors:** Gifta Evangeline Henry, Abishek Kumaresan, Swaminathan Angeline Vedha

**Affiliations:** a PG & Research Department of Chemistry, Bishop Heber College (Autonomous) Affiliated to Bharathidasan University Tiruchirappalli-620017 Tamil Nadu India angeline.vedha@gmail.com

## Abstract

In water treatment the formation of free available chlorine (FAC) during chlorine dioxide (ClO_2_) oxidation influences the overall formation of chlorine-containing by-products. However, the molecular factors governing FAC formation and the role of molecular structure in determining the oxidation pathway remain poorly understood. In this study, we employed density functional theory (DFT) to investigate the ClO_2_-mediated oxidation of three dihydroxybenzenes, catechol (CAT), resorcinol (RES), and hydroquinone (HYQ). Two competing pathways, consecutive hydrogen atom transfer (HAT/HAT) and hydrogen atom transfer followed by carbon-radical addition (HAT/CRA), were identified for CAT and HYQ. The HAT/HAT pathway is kinetically preferred for both compounds based on the calculated activation barriers. In contrast, RES undergoes competing HAT/CRA pathways involving *ortho*- and *para*-carbon attack, followed by HOCl formation. Quantum Theory of Atoms in Molecules (QTAIM) analysis reveals that different transition-state interactions contribute to the energetics of the competing pathways. Time Dependent Density Functional Theory (TDDFT) calculations additionally show that the oxidation intermediates exhibit enhanced absorption in the UV-visible region compared with the parent dihydroxybenzenes. Overall, the results demonstrate that the hydroxyl substitution pattern influences the competition between HAT and CRA and the associated chlorine-containing products formed during ClO_2_ oxidation.

## Abstract 2

DFT reveals competing HAT and CRA pathways in ClO_2_ oxidation of dihydroxybenzenes. The hydroxyl positioning controls pathway preference, reaction kinetics, and FAC formation.

## Graphical abstract

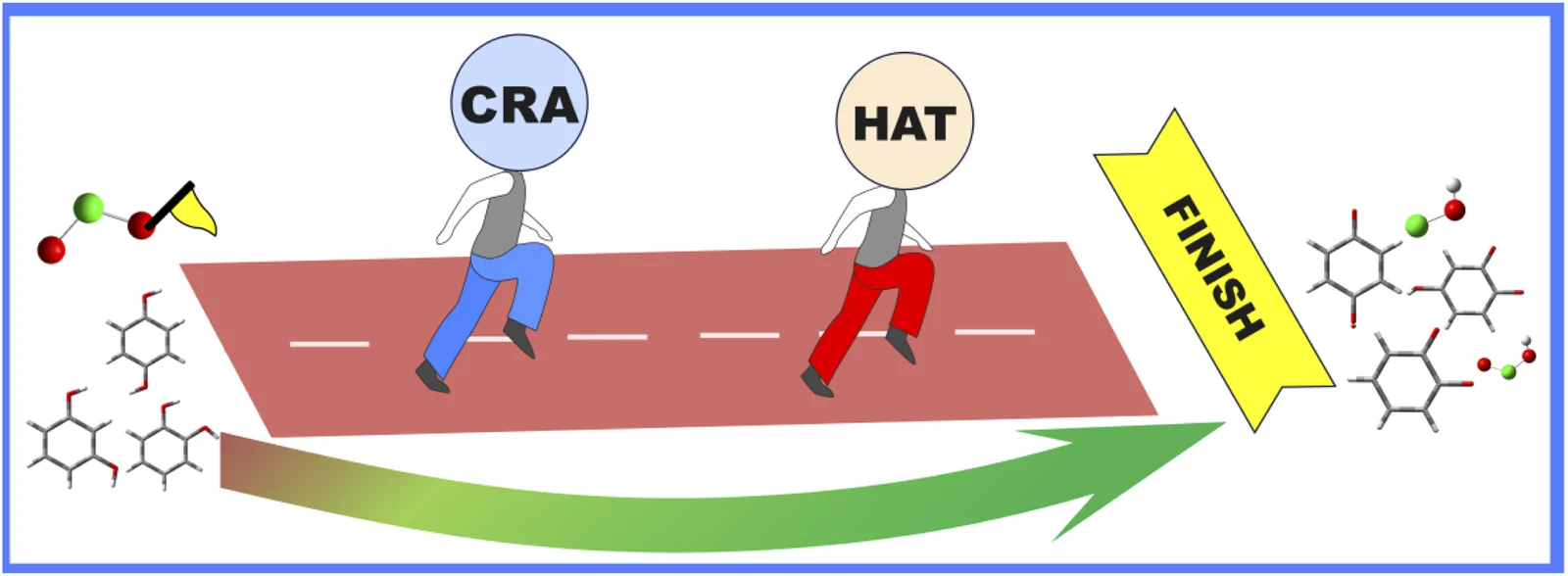

## Introduction

The formation of disinfection byproducts (DBPs), such as trihalomethanes (THMs) and haloacetic acids (HAAs), during chlorine-based water treatment processes has raised environmental and public health concerns due to their potential adverse health effects and the resulting regulatory restrictions on drinking water quality.^1–3^ In this context, chlorine dioxide (ClO_2_) has emerged as an alternative oxidant and disinfectant for water treatment applications.^4–11^ Unlike free chlorine, ClO_2_ exhibits high selectivity toward electron-rich organic moieties and does not readily participate in electrophilic substitution reactions, thereby minimizing the formation of halogenated DBPs.^12–18^ ClO_2_ predominantly reacts through one-electron transfer pathways and readily oxidizes phenols, anilines, and olefins.^13,19–22^ Consequently, ClO_2_-based oxidation has been adopted as a promising strategy for mitigating DBP formation during water treatment.^23,24^

Among the various electron-rich micropollutants in aquatic environments, phenolic compounds remain a persistent class of contaminants with reported concentrations in industrial effluents approximately 7000 mg L^−1^.^25–27^ In particular, dihydroxybenzenes comprising catechol (CAT), resorcinol (RES), and hydroquinone (HYQ) are frequently detected in industrial and municipal wastewaters. Notably, catechol and resorcinol concentrations of approximately 5500 and 250 mg L^−1^, respectively, have been reported in low-temperature coal-carbonization wastewater.^28^ CAT, RES and HYQ are commonly formed during the biodegradation of aromatic pollutants and exhibits high environmental reactivity, have been reported to be toxic toward aquatic organisms and are recognized as priority environmental pollutants in several regulatory frameworks.^26,29,30^ These concerns emphasize the need for effective treatment strategies for dihydroxybenzene-containing wastewaters. Hence ClO_2_ oxidation of CAT, RES and HYQ is studied in this work. In addition to the expected inorganic byproducts chlorite (ClO_2_^−^) and chlorate (ClO_3_^−^), ClO_2_ oxidation can generate significant amounts of free available chlorine (FAC) *in situ*.^31–34^ Since FAC acts as a secondary oxidant which inturn is capable of producing halogenated DBPs with natural organic matter,^35^ understanding the factors governing FAC generation is essential for assessing the overall environmental impact of ClO_2_-based treatment. Although ClO_2_ oxidation has been investigated widely, the influence of substituent on FAC formation and the mechanistic pathways remains insufficiently understood.

Previous experimental studies have provided valuable information regarding reaction kinetics and product distributions during ClO_2_ oxidation of phenolic compounds.^31,32,36–38^ However, elucidating these mechanistic pathways is important for optimizing ClO_2_-based oxidation processes and evaluating their implementation at larger treatment scales. Experimental observations have shown that under similar reaction conditions oxidation of CAT and HYQ produces negligible FAC, whereas RES generates ∼48% FAC.^32^ The observed FAC yield indicates that an HOCl-forming pathway contributes to RES oxidation. The experimental study also reported ClO_2_^−^ as the major non-FAC chlorine product (50 ± 0%), while Cl^−^ and ClO_3_^−^ were detected at approximately 1% each. The formation of ClO_2_^−^ can be attributed to the HClO_2_ generated during the initial HAT step, which subsequently dissociates to H^+^ and ClO_2_^−^. (i) How does the ClO_2_-based advanced oxidation process (AOP) differ among CAT, RES, and HYQ; (ii) can differences in reaction pathways and intermediate reactivity influence the efficiency of the overall oxidation process; (iii) given that hydrogen atom transfer (HAT) has been identified as the initiation step in ClO_2_ oxidation of halogen-substituted phenols, does the presence of a second hydroxyl group introduce the possibility of a consecutive HAT pathway that can compete with ClO_2_ attack at the carbon-centred radical site; and (iv) what role does HAT play? To address these questions, the oxidation mechanisms of CAT, RES, and HYQ were investigated using density functional theory (DFT).

Our previous work^39^ proved that DFT is an effective approach for elucidating radical-mediated oxidation mechanisms and the associated electronic structure changes. In the present work, DFT calculations were employed to systematically investigate the ClO_2_-mediated oxidation pathways of dihydroxy benzene. Potential energy surface analysis, Quantum Theory of Atoms in Molecules (QTAIM) analysis, and time-dependent DFT (TDDFT) calculations were carried out to elucidate the factors governing pathway selectivity and FAC formation. Collectively, these analyses reveal how hydroxyl substitution pattern controls the energetics, reactivity, and product distribution of ClO_2_-mediated oxidation in dihydroxybenzene systems.

## Theoretical methods

All quantum chemical calculations were carried out using the Gaussian 09W software package.^40^ In our previous investigation of ClO_2_-mediated oxidation of substituted phenols,^39^ ωB97XD was found to provide a consistent description of the reaction energetics. Accordingly, the ωB97XD/6-311+G(d,p)^41^ level of theory was adopted in the present study for consistency with our previous work. The calculated energetic trends obtained for the dihydroxybenzene systems were also consistent with the mechanistic trends established in our previous work,^39^ supporting the applicability of this computational approach to the present ClO_2_-mediated oxidation pathways. The long-range correction and empirical dispersion incorporated in ωB97XD further make it suitable for describing the radical species and noncovalent interactions encountered along these reaction pathways.^42–44^

Frequency calculations were performed at the same level of theory to characterize all stationary points on the potential energy surface. Minima were confirmed by the absence of imaginary frequencies, whereas each transition state exhibited a single imaginary frequency corresponding to the desired reaction coordinate. The *XYZ* coordinates of all the species in the present study are given in SI Table S7. All calculations were performed at pH 7 using the neutral substrate forms. The radical intermediates and TS1 were treated as doublet states using an unrestricted wavefunction, while the transition states were confirmed by the presence of one imaginary frequency. The charge, multiplicity, 〈*S*^2^〉 values, frequency data and IRC results are provided in the SI Table S3 and Fig. S6. Intrinsic reaction coordinate (IRC) calculations were subsequently carried out to verify the connectivity between transition states and their associated reactants and products. Solvent effects were incorporated throughout the calculations using the integral equation formalism polarizable continuum model (IEFPCM),^45^ with water representing the bulk aqueous medium. IEFPCM and related continuum approaches have been widely used for modelling solution-phase structures, energetics and reaction mechanisms.^51–53^ In particular, IEFPCM water has been applied in DFT studies of phenolic systems, including resorcinol, where both continuum and explicit-water models were examined.^54^ In the present study, the same implicit water treatment was applied consistently to CAT, RES and HYQ so that the energetic and mechanistic trends among the three systems could be compared under the same solvent description. However, IEFPCM does not usually account for specific water–solute hydrogen-bonding interactions. To obtain insight into the electronic factors governing the reaction mechanism, Quantum Theory of Atoms in Molecules (QTAIM) and Non-Covalent Interaction (NCI) analyses were performed for the optimized transition-state structures. Wavefunction files generated at the ωB97XD/6-311+G(d,p) level were analyzed using AIMAll Professional^46^ and Multiwfn software packages,^47^ while molecular visualizations were produced using VMD.^48^

The Gibbs free energy change (Δ*G*), activation free energy (Δ*G*^‡^), and enthalpy change (Δ*H*) for each elementary step was calculated according to the eqn (1)–(3)

1 Δ*G* = *G*(product complex) − *G*(reactant complex)

2 Δ*G*^‡^ = *G*(transition state) − *G*(previous minimum)

3 Δ*H* = *H*(product complex) − *H*(reactant complex)

Rate constants for the elementary reaction steps were estimated using transition-state theory through the Eyring–Polanyi equation:^49^

4 *k* = (*k*_B_*T*/*h*)exp(−Δ*G*^‡^/*RT*)

where, *k*_B_ is the Boltzmann constant, *h* is Planck's constant, *T* is the absolute temperature, *R* is the universal gas constant, and Δ*G*^‡^ represents the activation free energy.^50^ The calculated thermodynamic and kinetic parameters for all stationary points are summarized in Tables S2 and S3.

## Result and discussion

Experimental studies by Lutze *et al.*^32^ reported contrasting FAC yields for the three dihydroxybenzene isomers, with negligible FAC formation for CAT and HYQ and approximately ∼48% for RES. Since FAC formation is associated with HOCl release, the observed FAC yields indicate that HOCl formation contributes to RES oxidation. However, negligible FAC formation in CAT and HYQ does not by itself distinguish between the HAT/HAT and HAT/CRA pathways, as both pathways can lead to HClO_2_ formation. Accordingly, following the common initial HAT step, two competing pathways were considered: a second HAT (HAT/HAT) and carbon-radical addition (HAT/CRA) (Schemes 1–3).

**Scheme 1:**
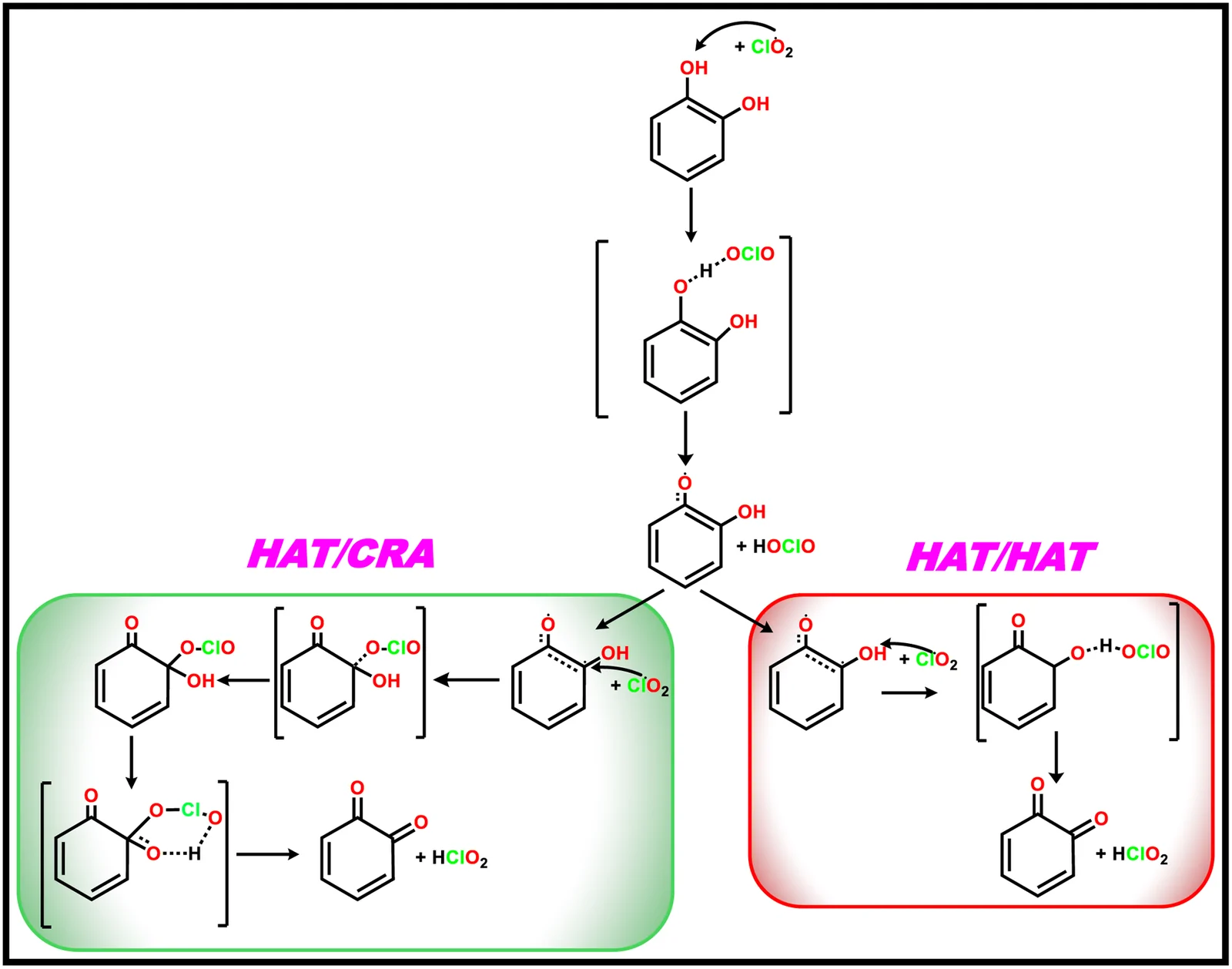
Proposed reaction mechanism of ClO_2_ with CAT.

**Scheme 2:**
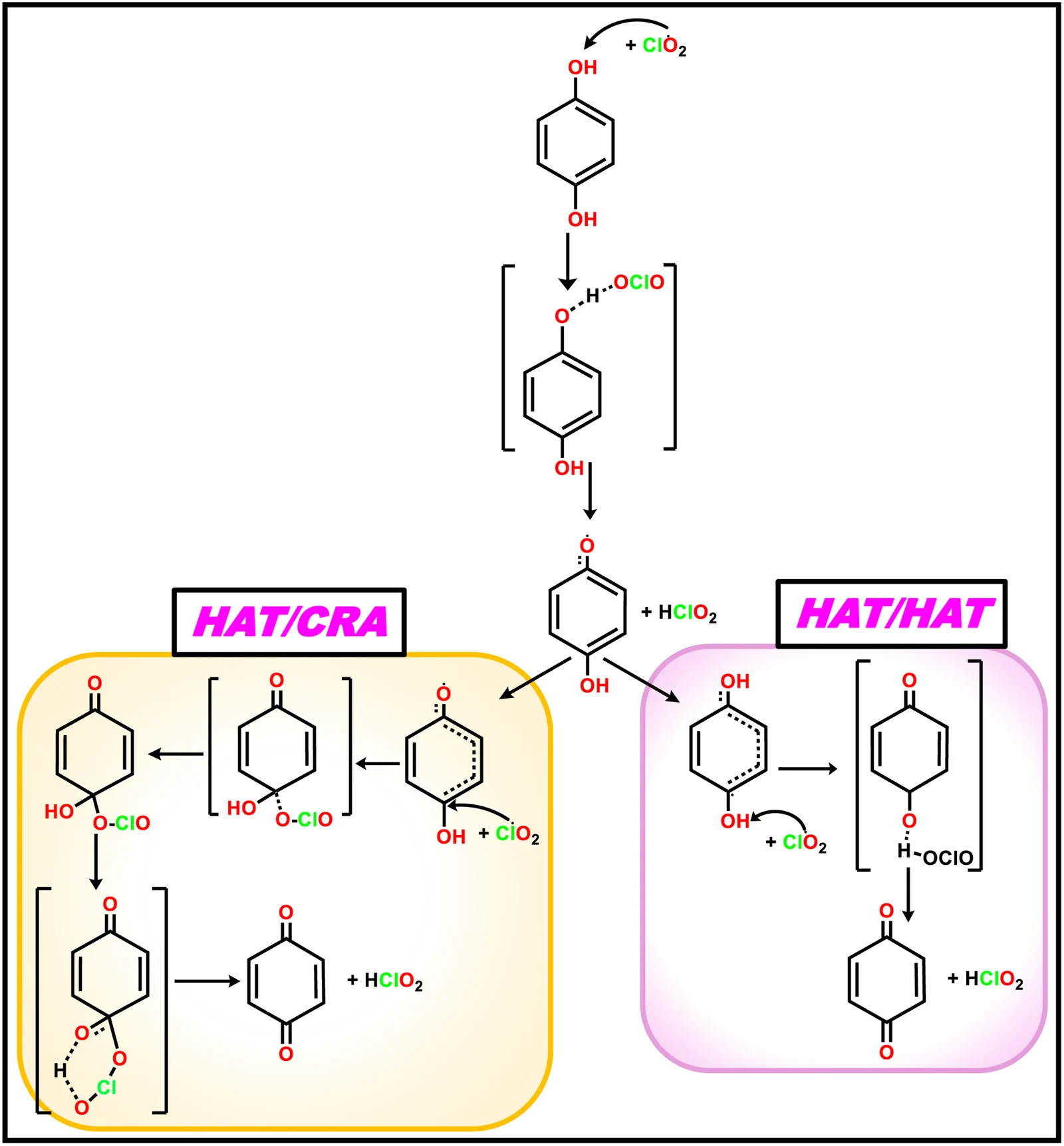
Proposed reaction mechanism of ClO_2_ with HYQ.

**Scheme 3:**
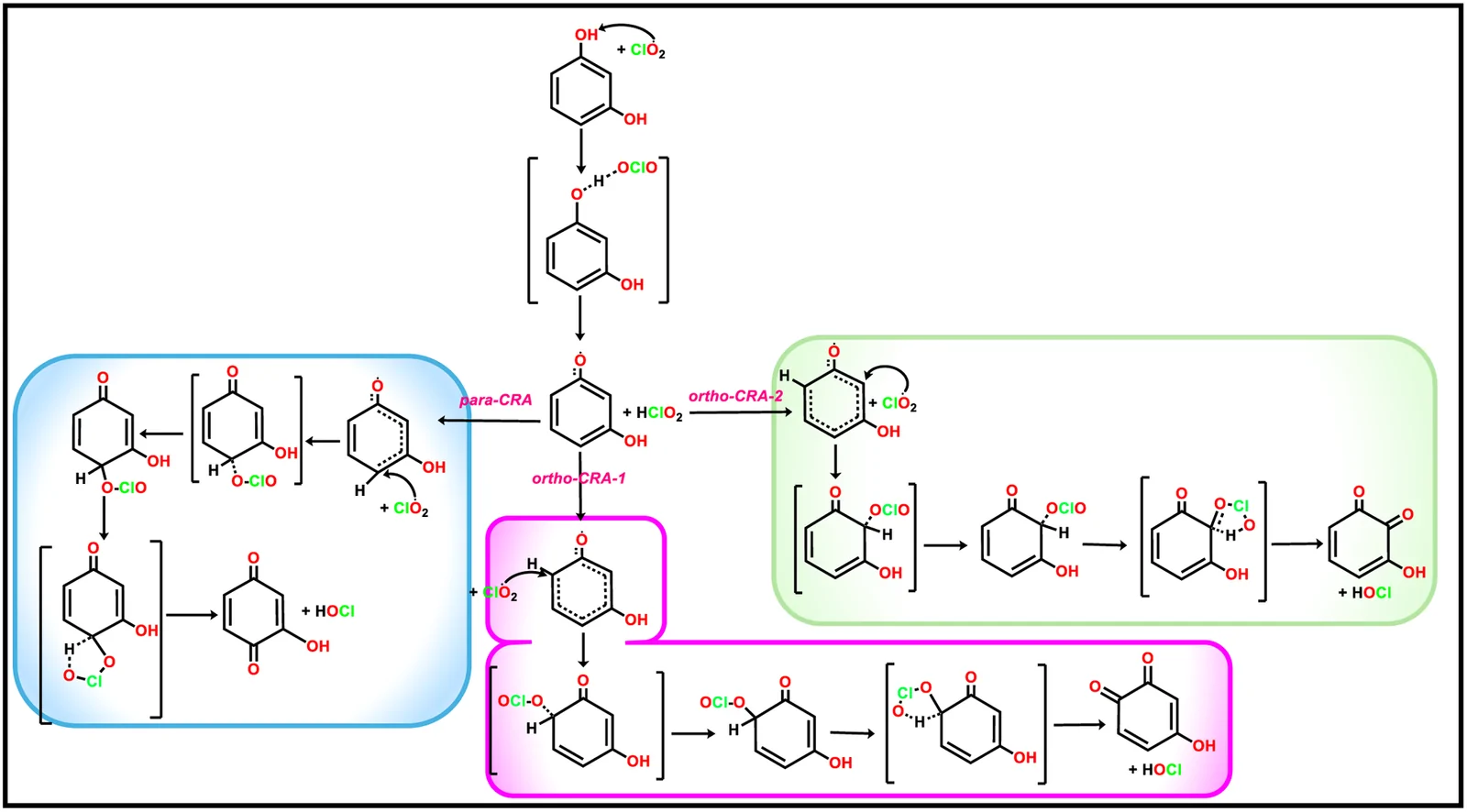
Proposed reaction mechanism of ClO_2_ with RES.

### Step (i) – hydrogen atom transfer

The initial step involves abstraction of the phenolic O–H by ClO_2_ through hydrogen atom transfer (HAT) followed by the formation of HClO_2_. HClO_2_ further dissociates into H^+^ and ClO_2_^−^. This elementary step is common to both reaction pathways. The first transition state (TS1) structure consists of H–O bond formation with the incoming ClO_2_ radical and O–H bond cleavage results in the generation of a phenoxide radical intermediate (SI Fig. S1).

The activation barriers for the initial HAT follows the order RES > CAT > HYQ. Although the primary HAT geometries are similar, differences in the electronic polarization of the HAT centre distinguish the three systems. In RES, the transferring H carries a lower positive charge (+0.492) and the accepting ClO_2_ oxygen is considerably less negative (−0.724) than in CAT (+0.505 and −0.823) and HYQ (+0.508 and −0.827), indicating less favourable polarization for hydrogen transfer. This distinct charge distribution can be associated with the *meta* arrangement of the second hydroxyl group in RES, which does not provide the same resonance interaction as CAT and HYQ. For CAT and HYQ, the charge distributions are nearly identical; however, the adjacent hydroxyl group in CAT creates an additional close O⋯O interaction (*ρ* = 0.0083 a.u.) together with the C⋯Cl interaction. For this O⋯O interaction, the local kinetic and potential energy densities are *G* = 0.006591 and *V* = −0.0063 a.u., respectively, giving *H*(*r*) = *G* + *V* = +0.000647 a.u. and |*V*|/*G* = 0.9063 (ref. 51 and 52) (see SI Fig. S4). These values are consistent with a weak closed-shell interaction and indicate that the O⋯O contact is less stabilizing than a shared-shell interaction. The close O⋯O contact arises from the *ortho* arrangement of the hydroxyl groups and is associated with greater steric constraint around the reacting O–H group, making the approach of ClO_2_ less favorable. In contrast, HYQ exhibits only the C⋯Cl secondary interaction and provides a less congested environment around the reacting hydroxyl group. The calculated steric strain energies of the corresponding transition states are 9.03 kcal mol^−1^ for CAT and 4.26 kcal mol^−1^ for HYQ, respectively, further supporting the greater structural constraint in CAT. The topological parameters at the bond critical point (BCP) for the transition-state structures are summarized in SI Table S6. A continuous basin path is observed along the O–H⋯O reaction coordinate through the H⋯O bond critical point, indicating the developing hydrogen-transfer interaction (Fig. 1). For HYQ-TS1, the H⋯O interaction has an electron density of 0.0583 a.u., a positive Laplacian of 0.1501 a.u. and −*G*/*V* = 0.82, with *H*(*r*) = −0.0105 a.u. These parameters indicate that the developing H⋯O interaction has partial shared character. The basin-path trajectory visualizes the electron-density pathway connecting the donor and acceptor atoms, while the Laplacian map shows the redistribution of electron density around the reactive region.

**Fig. 1:**
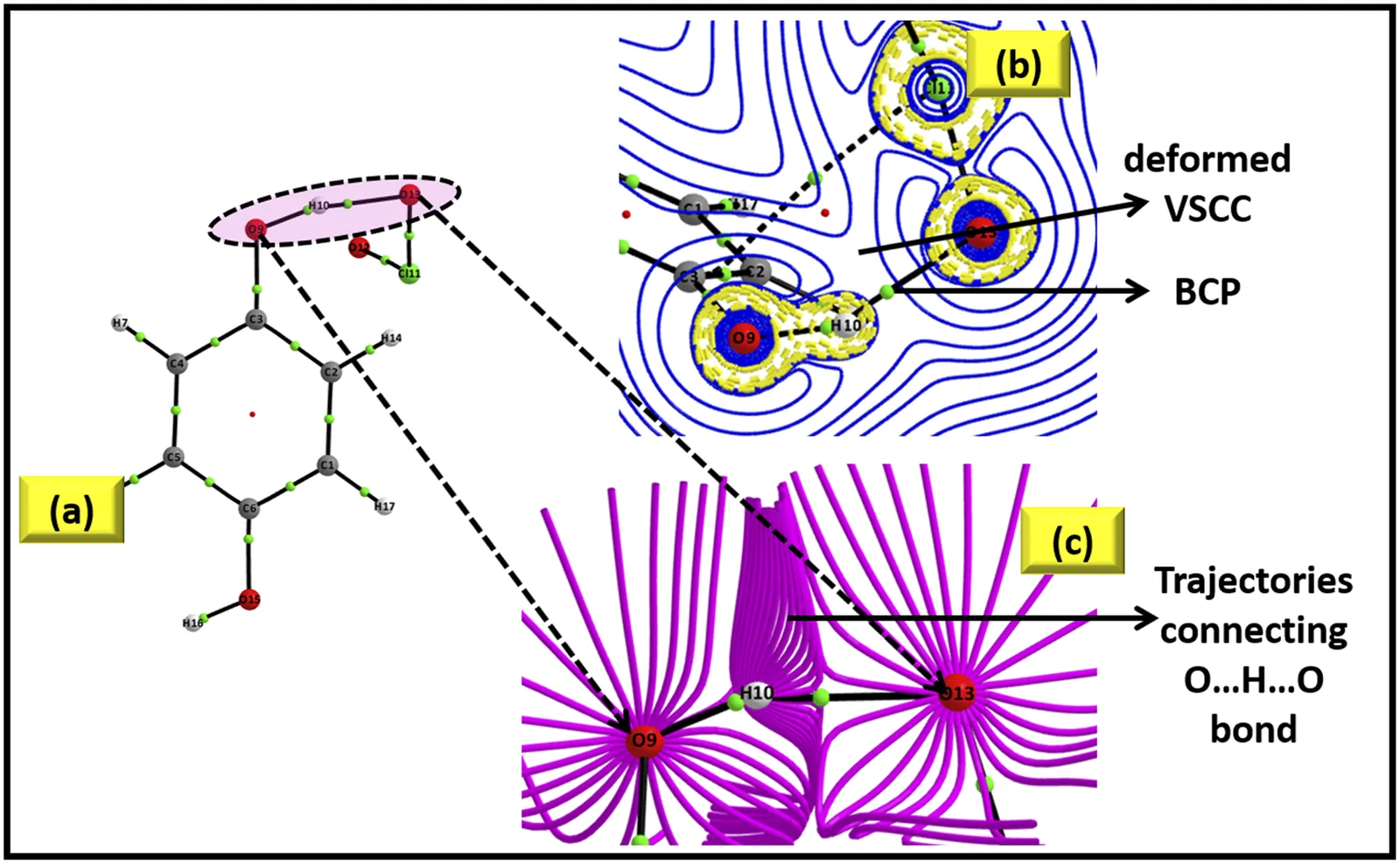
(a) QTAIM molecular graph of the TS1 in HYQ. (b) Laplacian of electron density contour map of the O–H⋯O reactive region illustrating distortion of the valence-shell charge concentration (VSCC) during hydrogen transfer. (c) Basin-path trajectory (pink) associated with the O⋯H⋯O.

### Reaction of ClO_2_ with catechol and hydroquinone

Following the initial HAT, the resulting radical intermediate presents two competing reaction possibilities for the second incoming ClO_2_ molecule: (i) carbon-radical attack (CRA) at a ring carbon possessing appreciable radical character (P-I) and (ii) abstraction of the remaining hydroxyl hydrogen through a second HAT (P-II). This provides a direct energetic basis for determining the preferred pathway. Under environmentally relevant conditions, the oxidation process proceeds through bimolecular interactions between phenolic substrates and ClO_2_ molecules, where the overall reaction rate depends on the concentrations of both reactants.

### Step (ii) – competition between HAT/CRA and HAT/HAT

Following the formation of the phenoxyl radical intermediate, the second ClO_2_ molecule can undergo either carbon-radical addition (CRA) or a second HAT. In the CRA pathway, ClO_2_ approaches the carbon centre bearing the radical through formation of a C⋯O bond, with corresponding distances of 2.61 and 2.59 Å for CAT and HYQ, respectively whereas, the competing HAT pathway proceeds through abstraction of the remaining hydroxyl hydrogen *via* an O–H⋯O interaction (SI Fig. S2). Comparison of the local activation barriers clearly favours HAT, with local activation barriers decreasing from 17.34 to 14.30 kcal mol^−1^ for CAT and from 16.85 to 11.68 kcal mol^−1^ for HYQ on moving from CRA to HAT (see Fig. 2 & 3). Moreover, HAT directly yields the corresponding benzoquinone and HClO_2_, whereas CRA requires an additional elimination step before product formation. Thus, the lower activation barrier together with the reduced number of elementary steps establishes consecutive HAT as the preferred oxidation route for both CAT and HYQ. Within the preferred HAT pathway (Fig. 4), HYQ exhibits a lower barrier (11.68 kcal mol^−1^) than CAT (14.30 kcal mol^−1^) and forms a more stabilized product (−16.03 *vs.* −21.41 kcal mol^−1^). This difference indicates that, although both positional isomers favour consecutive HAT, HYQ provides a more favourable energetic pathway than CAT.

**Fig. 2:**
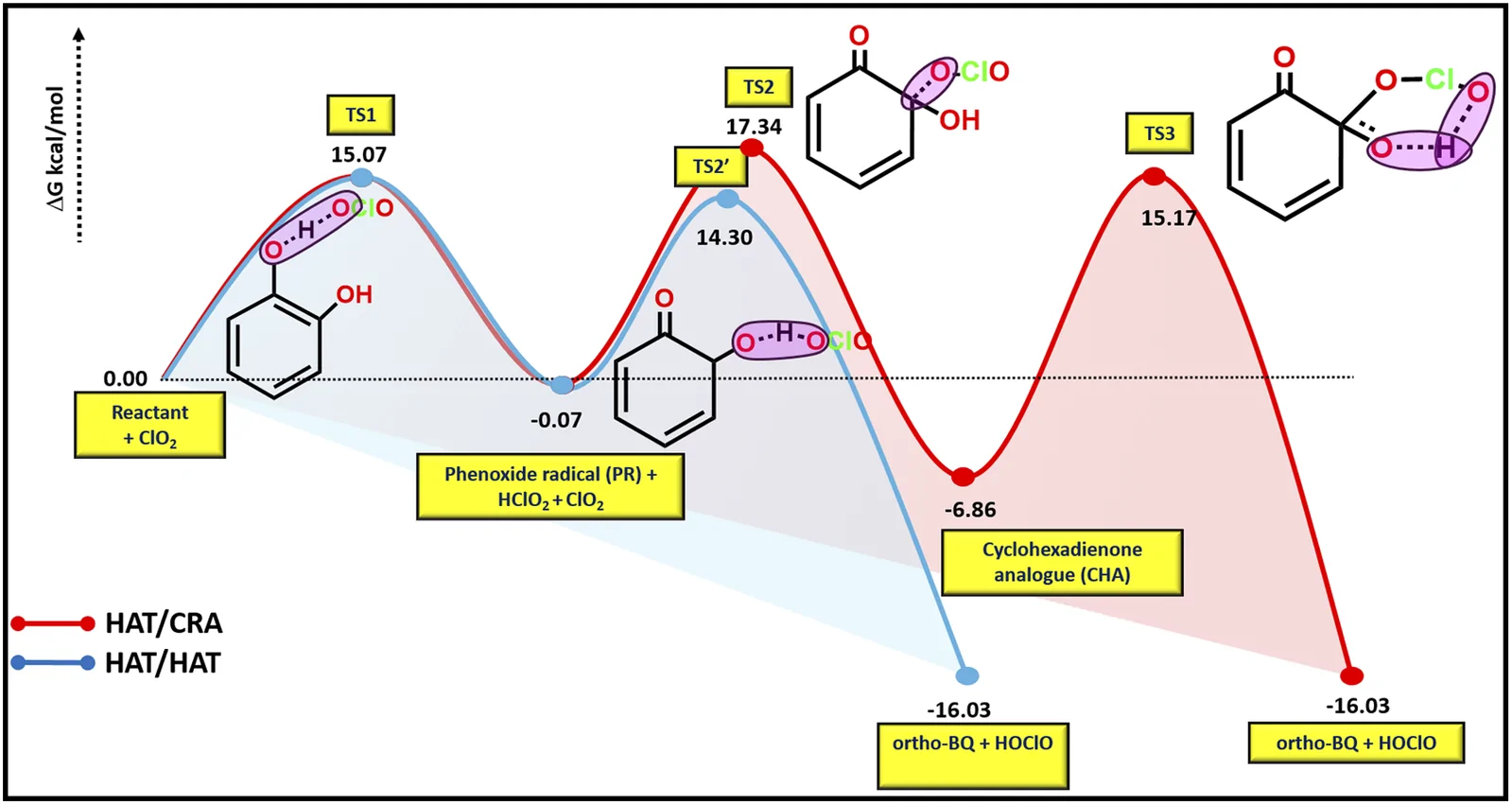
Free energy profiles for the oxidation of CAT by ClO_2_ through HAT/CRA and HAT/HAT.

**Fig. 3:**
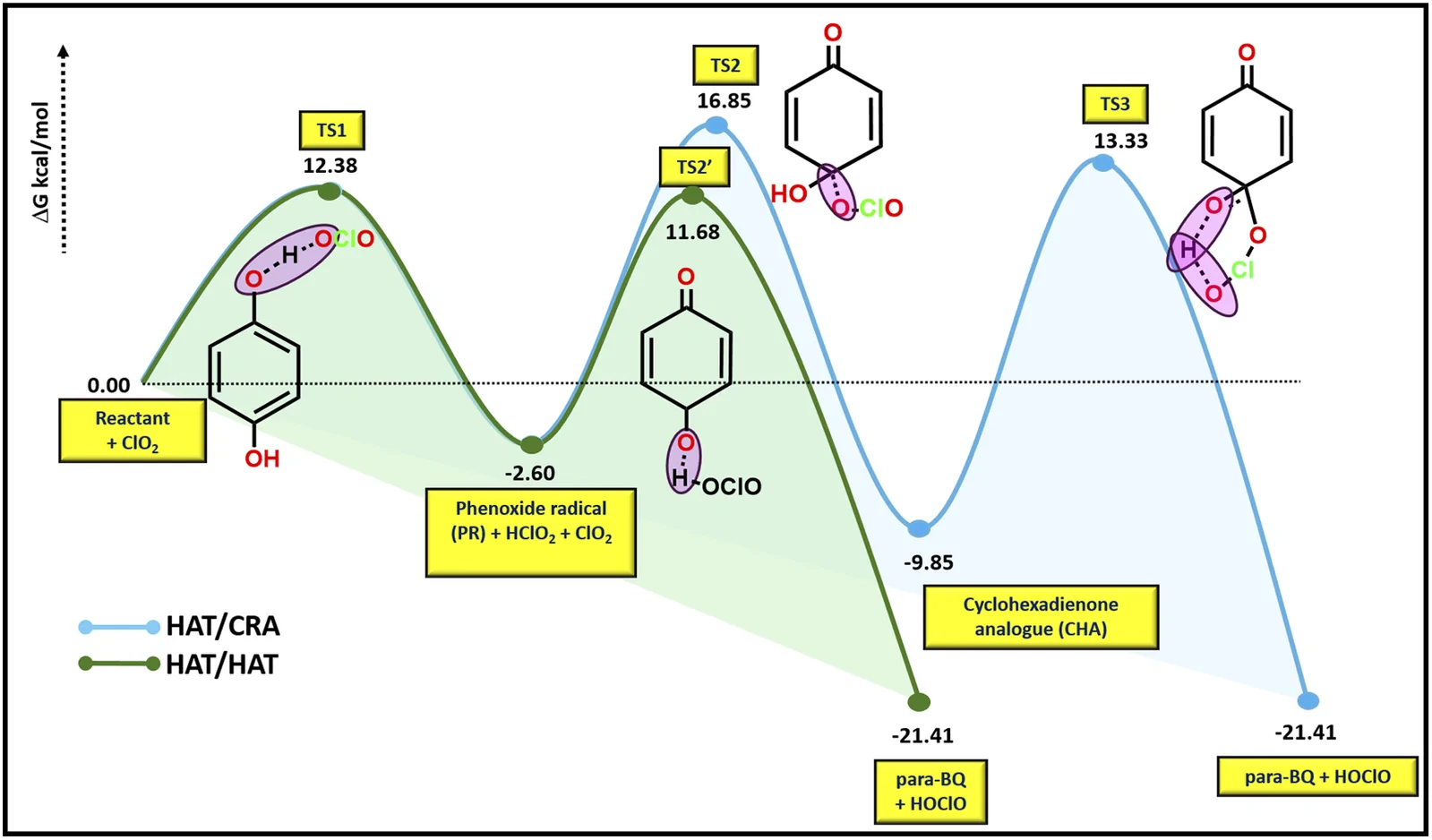
Free energy profiles for the oxidation of HYQ by ClO_2_ through HAT/CRA and HAT/HAT.

**Fig. 4:**
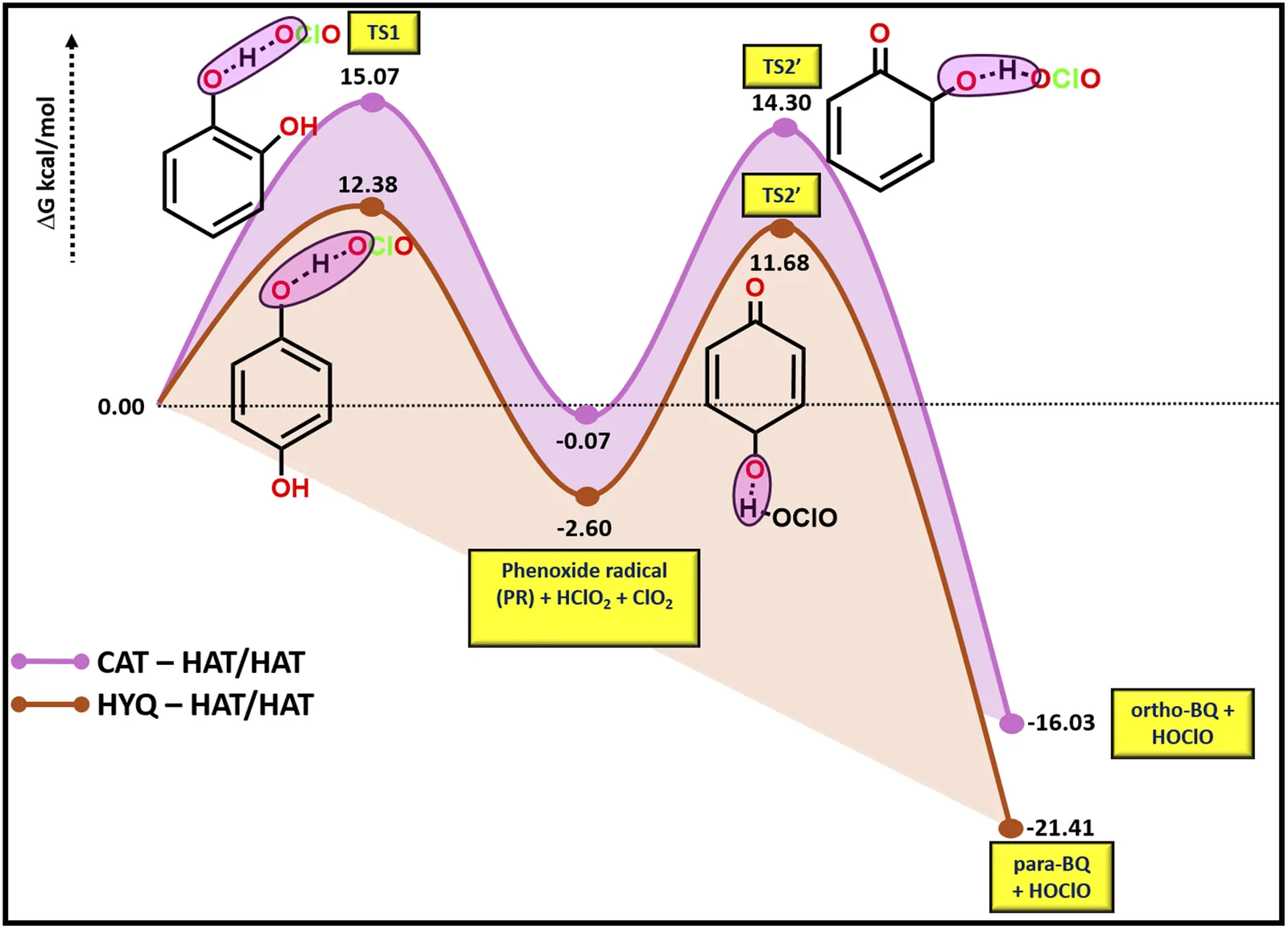
Comparison of free energy profiles in HAT/HAT pathway for CAT and HYQ.

QTAIM analysis further characterizes the second HAT transition state. For example, in HYQ-TS2′, the developing H⋯O interaction has an electron density of 0.1249 a.u., a positive Laplacian of 0.4888 a.u., −*G*/*V* = 0.54 and *H*(*r*) = −0.0722 a.u., indicating a relatively strong developing interaction with partial shared character. The basin-path trajectory in Fig. 5c shows the continuous O⋯H⋯O interaction through the H⋯O bond critical point, while the Laplacian map (Fig. 5b) illustrates the corresponding redistribution of electron density around the donor and acceptor atoms. These features support the development of the H⋯O interaction during the second HAT.

**Fig. 5:**
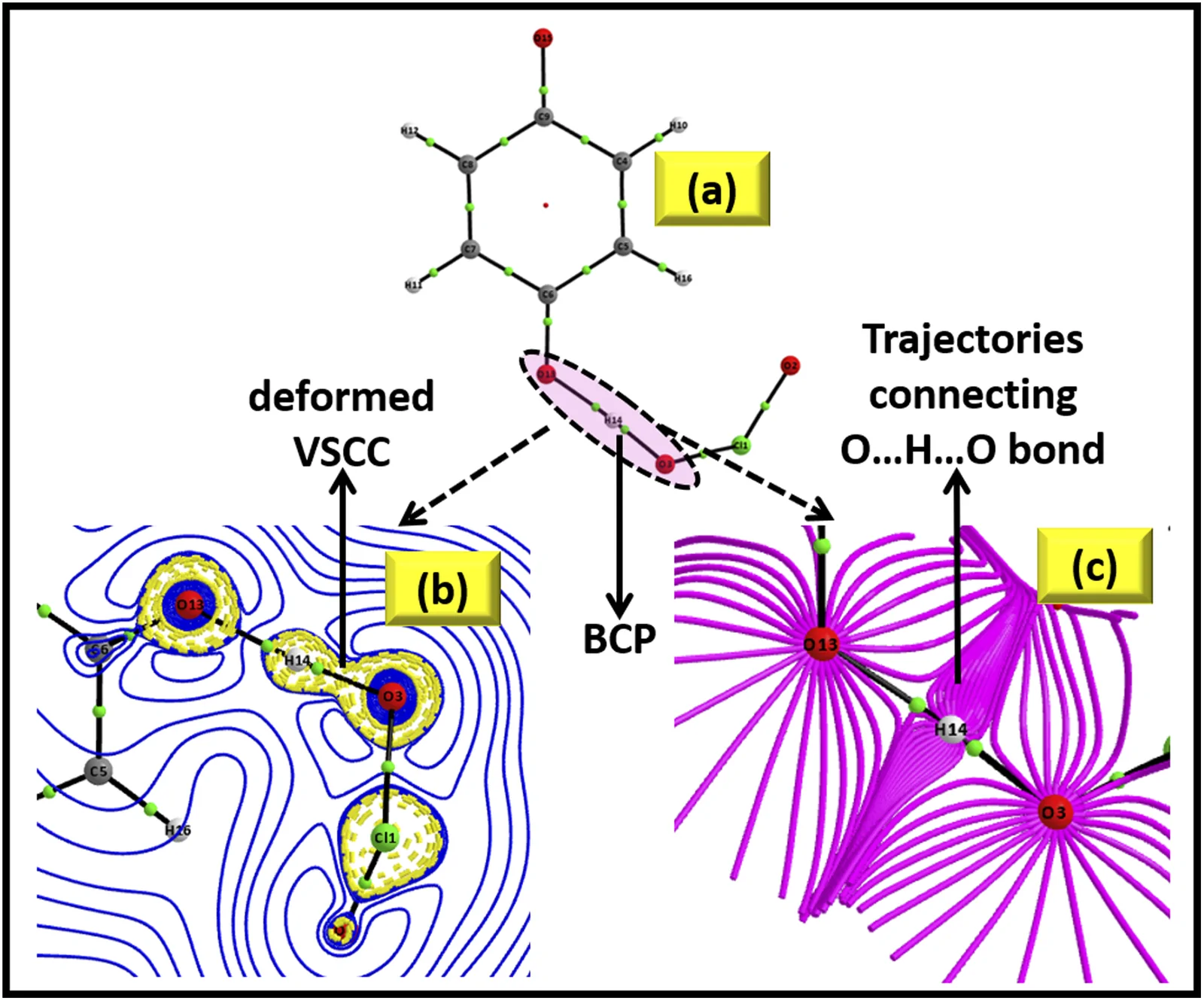
(a) QTAIM molecular graph of TS2′ in HYQ. (b) Laplacian of electron density contour map of the O–H⋯O reactive region illustrating distortion of the valence-shell charge concentration (VSCC) during hydrogen transfer. (c) Basin-path trajectory (pink) associated with the O⋯H⋯O bond.

### Step (iii) – release of HClO_2_

The CRA pathway subsequently proceeds through an additional elimination step involving an S(6) pseudo-cyclic transition-state motif, leading to HClO_2_ release and formation of *ortho*-benzoquinone (*o*-BQ) from CAT and *para*-benzoquinone (*p*-BQ) from HYQ (SI Fig. S3). CAT exhibits a higher local activation barrier of 15.17 kcal mol^−1^ compared with 13.33 kcal mol^−1^ for HYQ, while the corresponding product energies are −16.03 and −21.41 kcal mol^−1^, respectively. Thus, the elimination step is both kinetically and thermodynamically more favourable for HYQ than for CAT. In both systems, the transition state is stabilized by an O⋯H interaction within the S(6) motif; however, the difference in activation barriers cannot be explained solely by the strength of this interaction, as further examined through QTAIM analysis. Results show that the transition state has a well-developed O⋯H interaction within the S(6) motif with slight covalent characters (−*G*/*V* = 0.71). This stabilizes of the cyclic transition state during the concerted bond-breaking and bond-forming process. However in CAT the adjacent carbonyl oxygen introduces an additional close O⋯O contact (3.32 Å), resulting in a higher activation barrier relative to HYQ.

### Reaction of ClO_2_ with resorcinol

Unlike CAT and HYQ, RES has been experimentally reported to produce approximately ∼48% FAC, providing evidence for the formation of HOCl during ClO_2_ oxidation. After the initial HAT step, the remaining hydroxyl group in RES is *meta* to the principal radical-delocalization positions, and therefore the resonance arrangement that facilitates the consecutive HAT pathway in CAT and HYQ is not available. Nevertheless, the competing HAT/HAT pathway was also evaluated for RES to assess whether it could provide an alternative non-FAC-forming route. The calculated HAT/HAT pathway has a local activation barrier of 23.56 kcal mol^−1^, while the resulting *meta*-benzoquinone product lie at 26.66 kcal mol^−1^. The high energy of the product indicates that *meta*-benzoquinone is highly unstable and cannot be isolated hence, this pathway is highly unfavourable and may allow the system to return toward the preceding phenoxy-radical intermediate. The corresponding energy profile is provided in the SI. Thus, the present mechanistic investigation focused on HOCl-forming pathways for RES. Spin-density and condensed Fukui-function analyses of the RES radical intermediate reveal the *ortho* and *para* carbon atoms as the predominant reactive sites, whereas the *meta* carbon shows comparatively lower local reactivity (SI Table S4). Accordingly, CRA of the second ClO_2_ molecule was considered at the *para* position (P-III: *para*-CRA) and at the two *ortho* positions (P-IV: *ortho*-CRA-1 and *ortho*-CRA-2) (see Scheme 3). The calculated local activation barriers for the two *ortho* pathways are nearly identical (21.45 and 21.16 kcal mol^−1^), indicating that both *ortho* carbon atoms are kinetically accessible. However, *ortho*-CRA-1 affords a considerably more stabilized cyclohexadienone intermediate (−12.17 kcal mol^−1^) than *ortho*-CRA-2 (−9.13 kcal mol^−1^), suggesting that the *ortho* position opposite to the *meta*-hydroxyl substituent is thermodynamically preferred. Both pathways ultimately release HOCl and form hydroxyl benzoquinone products, providing a molecular-level explanation for the experimentally observed FAC generation (Fig. 6). The retention of an additional hydroxyl group in these products may provide a reactive site for subsequent oxidation, allowing further transformation beyond the initial ClO_2_-mediated oxidation step.

**Fig. 6:**
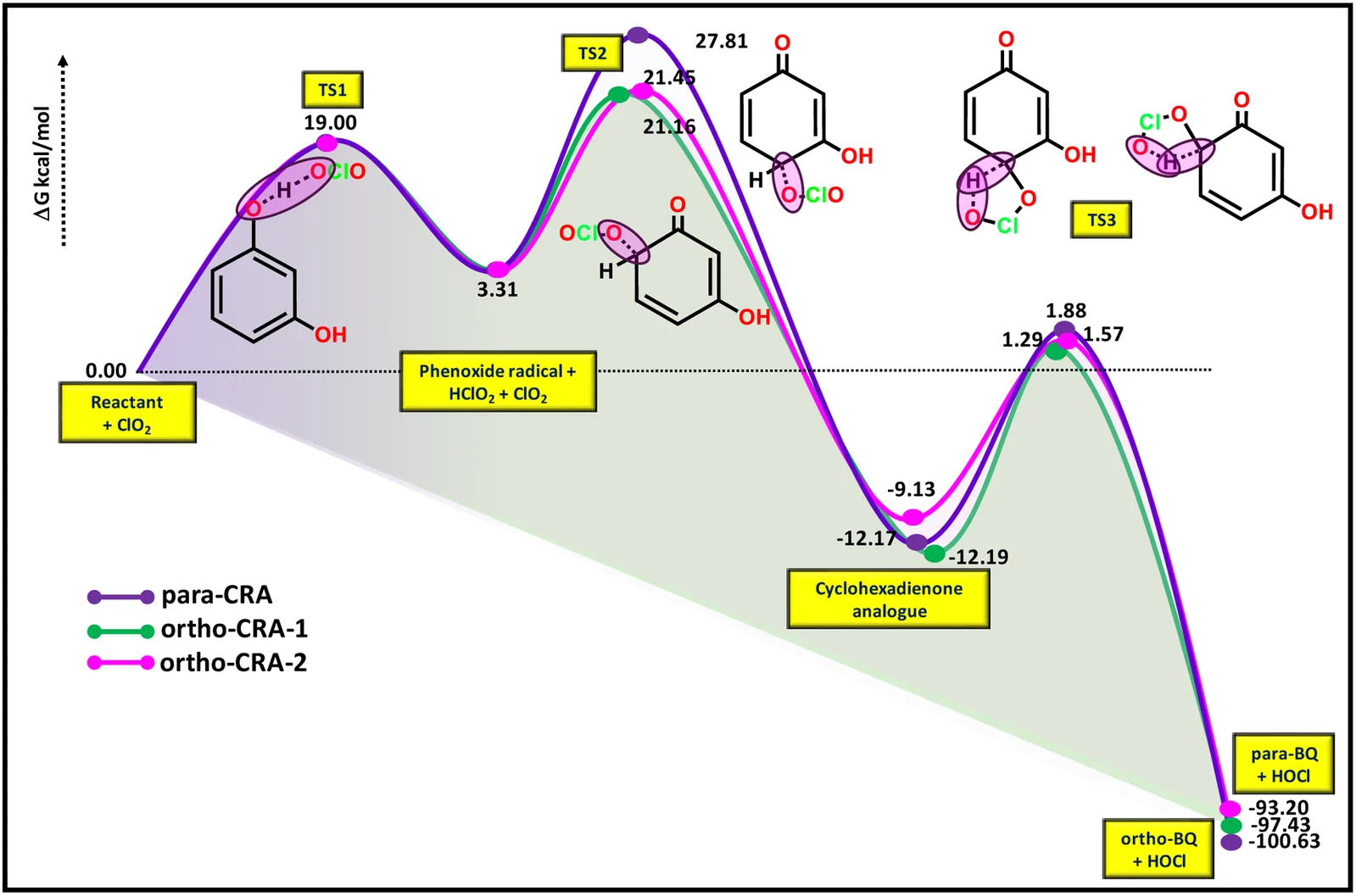
Free energy profiles for the oxidation of RES by ClO_2_ through *para*-CRA, *ortho*-CRA-1 and *ortho*-CRA-2.

From the energy profile it is evident that attack at the *para* carbon (*para*-CRA) ultimately leads to a *para*-benzoquinone-type product, whereas *ortho*-CRA-1, which is energetically preferred over *ortho*-CRA-2, yields the corresponding *ortho*-benzoquinone derivative. The major energetic difference between these pathways occurs during the step (ii), where P-III exhibits a local activation barrier ∼6 kcal mol^−1^ higher than P-IV. This difference indicates that, although the *para* carbon is predicted to possess greater local reactivity, the approach and addition of ClO_2_ at this site is kinetically less favourable than at the *ortho* position. In contrast, the subsequent HOCl-elimination barriers differ by only 0.59 kcal mol^−1^, indicating that this step is affected only marginally by the site of the preceding ClO_2_ addition. However, thermodynamically, the final products differ by ∼3 kcal mol^−1^, with the *para*-BQ type product being more stable. Thus, *ortho*-CRA-1 is kinetically favoured through its lower CRA barrier and greater intermediate stabilization, whereas *para*-CRA is thermodynamically preferred owing to the greater stability of the resulting product.

QTAIM analysis further characterizes the developing C⋯O interaction during the CRA step. For RES-TS2, the C⋯O interaction has an electron density of 0.0243 a.u. and −*G*/*V* = 1.12, indicating a weak closed-shell interaction. The basin-path trajectory in Fig. 7b visualizes the Laplacian of electron-density associated with the developing C⋯O interaction where, the valence-shell charge concentration (VSCC) of C is deformed towards the donor O. These topological features are consistent with the development of the C⋯O interaction during the CRA step. The QTAIM analysis of TS3 further characterizes the electronic changes accompanying HOCl elimination. The O⋯H interaction at RES-TS3 has a low electron density of 0.0241 a.u., a positive Laplacian of 0.0824 a.u. and −*G*/*V* = 1.15, consistent with a weak closed-shell interaction. The basin-path trajectories in Fig. 7d, further visualize the electron-density redistribution associated with O–C bond formation, respectively. The trajectory associated with the developing O–C interaction is directed toward the bond critical point, indicating formation of the new bond.

**Fig. 7:**
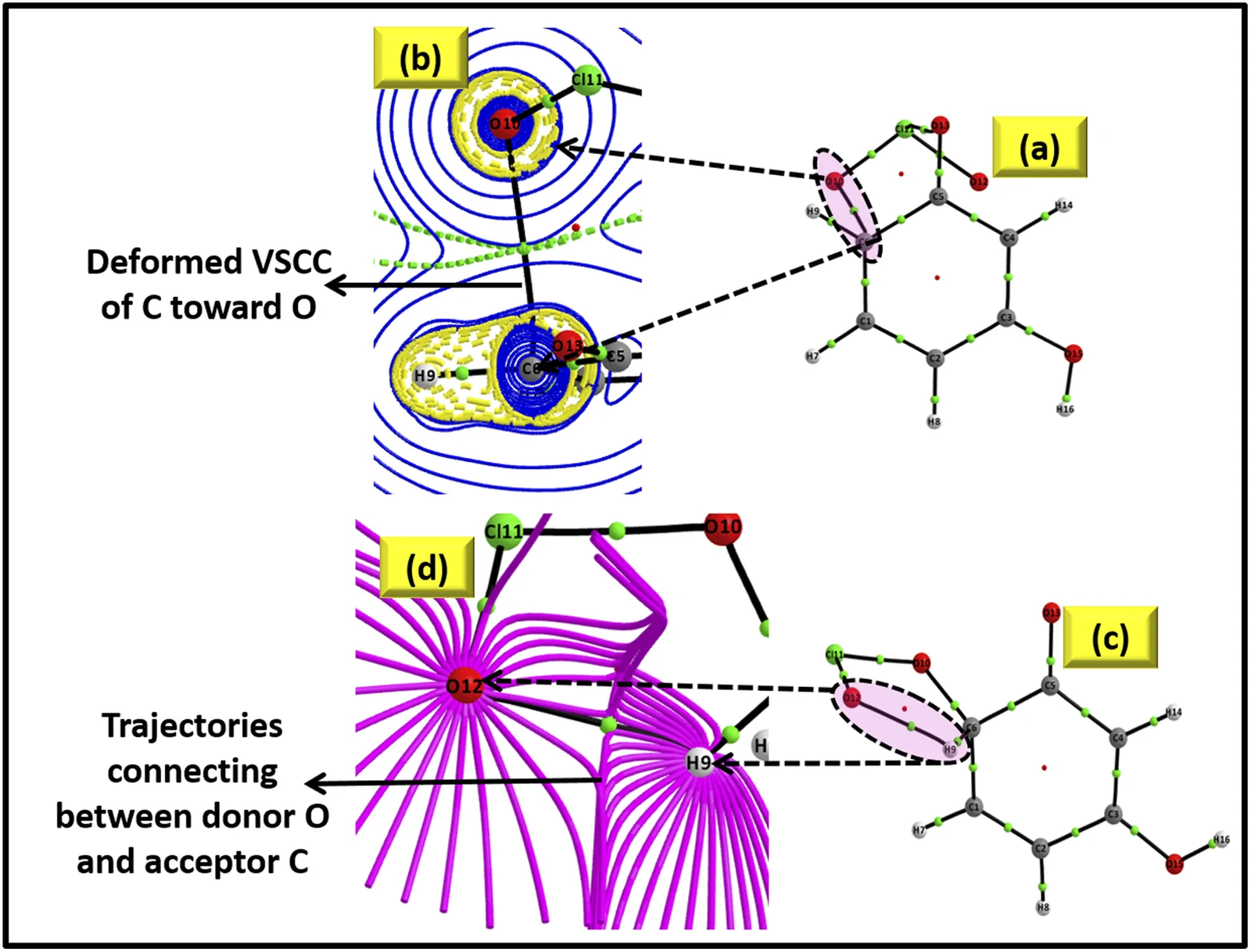
(a) QTAIM molecular graph of RES-TS2. (b) Laplacian of electron-density contour map showing deformation of the VSCC around the reacting C and O atoms. (c) Molecular graph of RES-TS3 and (d) basin-path trajectories showing the electron-density pathways connecting the donor O and acceptor C atoms during the developing C⋯O interaction.

### Kinetic analysis and structure–reactivity relationship

Kinetic analysis of CAT & HYQ reveal that in the HAT/CRA pathway step (ii) is rate determining step (RDS) with calculated rate constants of 1.19 and 2.73 M^−1^ s^−1^. In contrast, in the HAT/HAT pathway, the initial HAT (step (i)) is the RDS, with corresponding rate constants of 5.47 × 10 and 5.15 × 10^3^ M^−1^ s^−1^, while the second HAT proceeds more readily. The first HAT alters the electronic environment of the oxygen of the hydroxyl group in the resulting phenoxyl radical. NBO analysis shows that the charge on the hydroxyl oxygen changes from −0.720 to −0.647 in CAT and from −0.707 to −0.651 in HYQ, while the hydroxyl hydrogen remains positively polarized. This increase in positive charge facilitates the second HAT and formation of the quinone structure. In RES, both the *para*-attack (P-III) and *ortho*-attack (P-IV) pathways exhibit step (ii) as the RDS. The corresponding rate constants are 2.50 × 10^−8^ and 1.15 × 10^−3^ M^−1^ s^−1^ respectively, indicating that *ortho* attack is kinetically favoured. Overall, hydroxyl positioning determines both the preferred oxidation route and the rate-controlling elementary step during ClO_2_ oxidation (Table 1).

**Table 1:** Kinetic parameters at the RDS of all the species in the study^a^

Pathway	Δ*G*^‡^	*k*_RDS_
**CAT**		
HAT/CRA	17.34	1.19 × 10^0^
HAT/HAT	15.07	5.47 × 10^1^

**RES**		
*para*-CRA	27.81	2.50 × 10^−8^
*ortho*-CRA-1	21.45	1.15 × 10^−3^
*ortho*-CRA-2	21.16	1.88 × 10^−3^

**HYQ**		
HAT/CRA	16.85	2.73 × 10^0^
HAT/HAT	12.38	5.15 × 10^3^

a The units are in kcal mol^−1^ for Δ*G*^‡^ and M^−1^ s^−1^ for *k*_RDS_.

### TDDFT analysis of intermediates and products

TDDFT calculations were performed for the optimized parent compounds, oxidation intermediates and products to examine the changes in their electronic absorption following ClO_2_ oxidation. This analysis was performed to assess whether the oxidation products generated during ClO_2_ treatment could exhibit enhanced absorption at longer wavelengths and therefore be susceptible to subsequent UV/visible-light-induced transformation. The parent dihydroxybenzenes CAT, RES and HYQ exhibit their first electronic transitions in the UV region at 244.23, 241.97 and 263.98 nm, respectively. Following ClO_2_ oxidation, several intermediates and products exhibit pronounced red shifts, with absorption extending from the UV into the visible region (391.32–819.36 nm).

For example, CAT-PR, *o*-BQ and RES-PR exhibit calculated absorption maxima at 699.45, 557.69 and 819.36 nm, respectively. Similar red-shifted absorption of oxidation intermediates has been reported by Mai *et al.*,^53^ who used TD-DFT to assess the photolytic behaviour of intermediates and products formed during aniline oxidation and reported transitions extending into the visible region. The present results similarly indicate that ClO_2_ oxidation can generate intermediates and products with absorption extending into the visible region. This observation provides a computational basis for considering combined UV/ClO_2_ treatment as a potential approach for further transformation of oxidation intermediates in future water-treatment studies. Experimental studies will be required to determine whether the calculated visible-light absorption results in measurable photochemical transformation under water-treatment conditions. Representative absorption maxima are shown in Fig. 8, while the complete excitation energies and oscillator strengths are provided in the SI (Table S5).

**Fig. 8:**
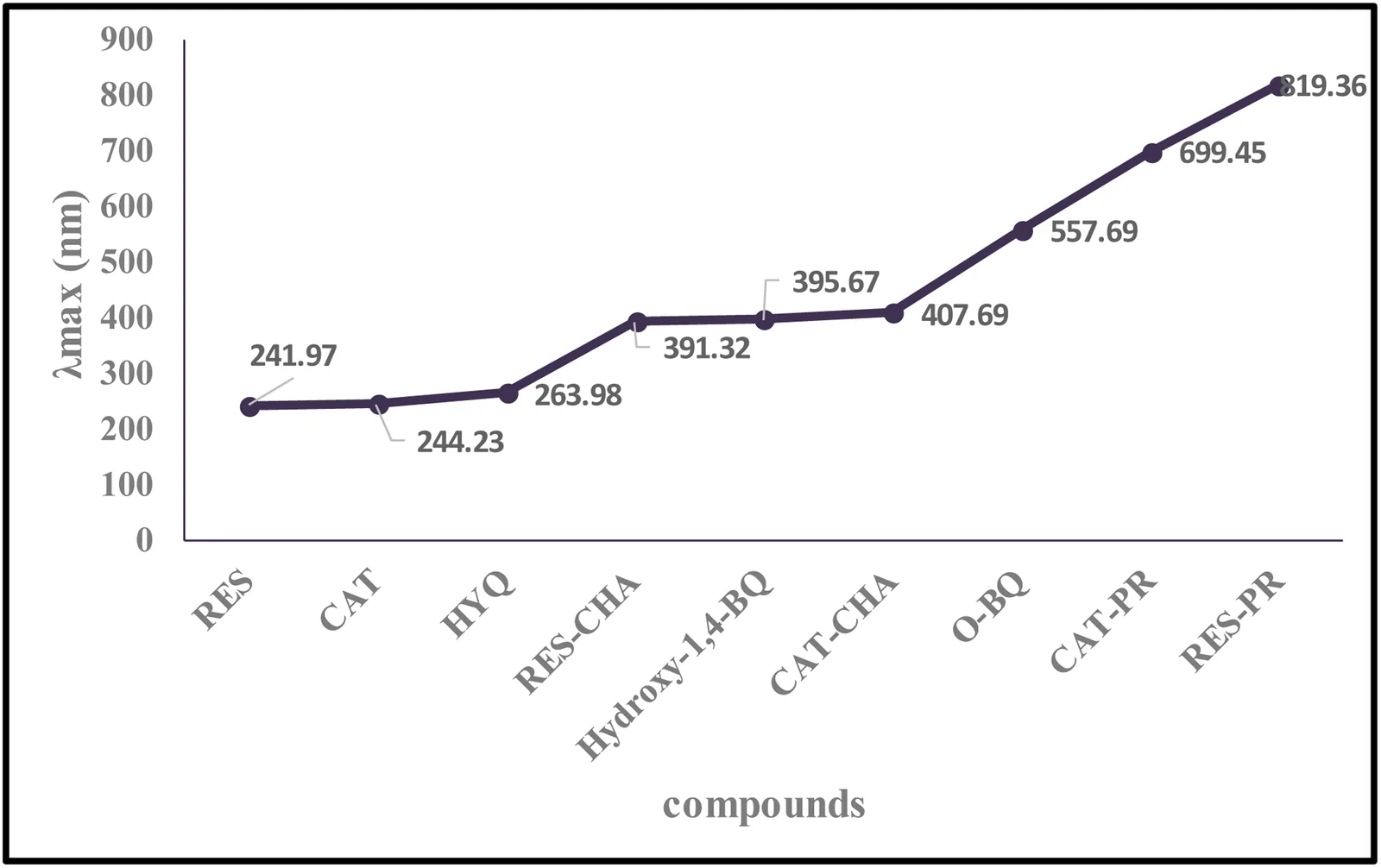
Calculated first electronic absorption wavelengths (*λ*_max_) of CAT, RES and HYQ and their corresponding ClO_2_ oxidation intermediates and products, illustrating the oxidation-induced red shift toward longer wavelengths.

## Conclusion

The present DFT investigation establishes that the hydroxyl substituent position is the primary factor governing the oxidation mechanism of dihydroxybenzenes by ClO_2_. For CAT and HYQ, the reaction preferentially proceeds through two successive hydrogen atom transfer (HAT/HAT), as it exhibits lower local activation barriers than the conventionally proposed radical-addition pathway HAT/CRA. Consequently, the direct formation of HClO_2_ in HAT/HAT explains the experimentally observed 0% FAC. In contrast, RES follows a different oxidation mechanism. After the initial HAT step, the second ClO_2_ molecule preferentially attacks the *ortho* and *para* radical centers. Fukui function analysis identifies the *para* carbon as the most reactive site, consistent with the thermodynamic stability, while the *ortho* pathway remains kinetically preferred. Both pathways ultimately eliminate HOCl, providing a possible mechanistic explanation for the experimentally observed 48% FAC formation during RES oxidation. Kinetic analysis further reveals that the C=O bond-forming step is the RDS for HAT/CRA pathway while the initial HAT step is the RDS in HAT/HAT pathway. QTAIM analysis shows that hydrogen-bond strength alone does not determine the activation barrier; the overall transition-state arrangement also contributes to pathway preference. TDDFT further indicates that the intermediates possess enhanced UV absorption compared to the parent dihydroxybenzenes, highlighting their potential for degradation in UV/ClO_2_ environment.

Overall, this work demonstrates that hydroxyl substitution pattern influences the competition between HAT and CRA during ClO_2_ oxidation and determines the chlorine-containing products accessible through the investigated pathways. For CAT and HYQ, the calculated HAT/HAT pathway is kinetically preferred, while both HAT/HAT and HAT/CRA pathways lead to HClO_2_ formation. For RES, the investigated *ortho*- and *para*-CRA pathways lead to HOCl formation and therefore provide possible mechanistic routes consistent with the experimentally observed FAC formation. These findings provide a mechanistic framework for predicting by-product formation during ClO_2_-based water treatment and offer molecular-level insight in designing oxidation processes with improved selectivity and reduced formation of undesirable chlorine-containing by-products.

## Author contributions

The manuscript was written through contributions of all authors. All authors have given approval to the final version of the manuscript. All authors contributed equally.

## Conflicts of interest

There are no conflicts to declare.

## Supplementary Material

RA-016-D6RA08249A-s001

## Data Availability

Supplementary information (SI): all optimized geometries (*XYZ* format), additional Fig. S1–S6, and Tables S1–S7. See DOI: https://doi.org/10.1039/d6ra08249a.
