# Supplementary material for: Competition between hydrogen atom transfer and carbon radical addition pathways in ClO2 oxidation of dihydroxybenzenes for water treatment: a DFT study
Source: RSC Adv. 2026 Oct 26. Online ahead of print. doi: 10.1039/d6ra08249a (PMC13697628; doi:10.1039/d6ra08249a)
Supplement: Supplementary file 1 [file RA-OLF-D6RA08249A-s001.pdf]

Supplementary Information (SI) for RSC Advances.
This journal is © The Royal Society of Chemistry

Supporting Document

Computational Insights into Chlorine-dioxide Mediated Advance Oxidation Process of Halogenated Phenols

Gifta Evangeline Henry [a] , *Abishek Kumaresan* [a], *Swaminathan Angeline Vedha*[a]

[a] PG & Research Department of Chemistry, Bishop Heber College Affiliated to Bharathidasan University, Tiruchirappalli – 620017, Tamil Nadu, India

email: angeline.ch@bhc.edu.in

Contents:

a) Figures:

S1: Optimized structures of TS1 with selective bond lengths and bond angles.

S2: Optimized structures of TS2 and TS2' with selective bond lengths and bond angles

S3: Optimized structures of TS3 and TS3' with selective bond lengths and bond angles

S4: QTAIM parameters at the O...O interaction in CAT TS1

S5: Free energy profile of the HAT/HAT pathway for RES.

S6: IRC graphs of all the transition state species identified in the present study

b) Tables:

S1: Gibbs free energy and enthalpy of ClO_2 and relevant by-products

S2: Gibbs free energy of all the species involved in the reaction

S3: Charge, spin multiplicity, number of imaginary frequencies, imaginary frequency values, and $\langle S^2 \rangle$ values for the optimized structures considered in the reaction pathways

S4: Condensed Fukui Function values of phenoxide radical of RES

S5: Excited state properties of all the reactants, intermediates and products included in the reaction

S6: Topological parameters at the BCP for TS1, TS2, TS2' TS3 and TS3' computed at the same level of theory

S7: XYZ coordinates of all the structures included in this study.

Figure S1: Optimized structures of TS1 with selective bond lengths and bond angles calculated at the wB97XD/6-311+G(d,p) level of theory.

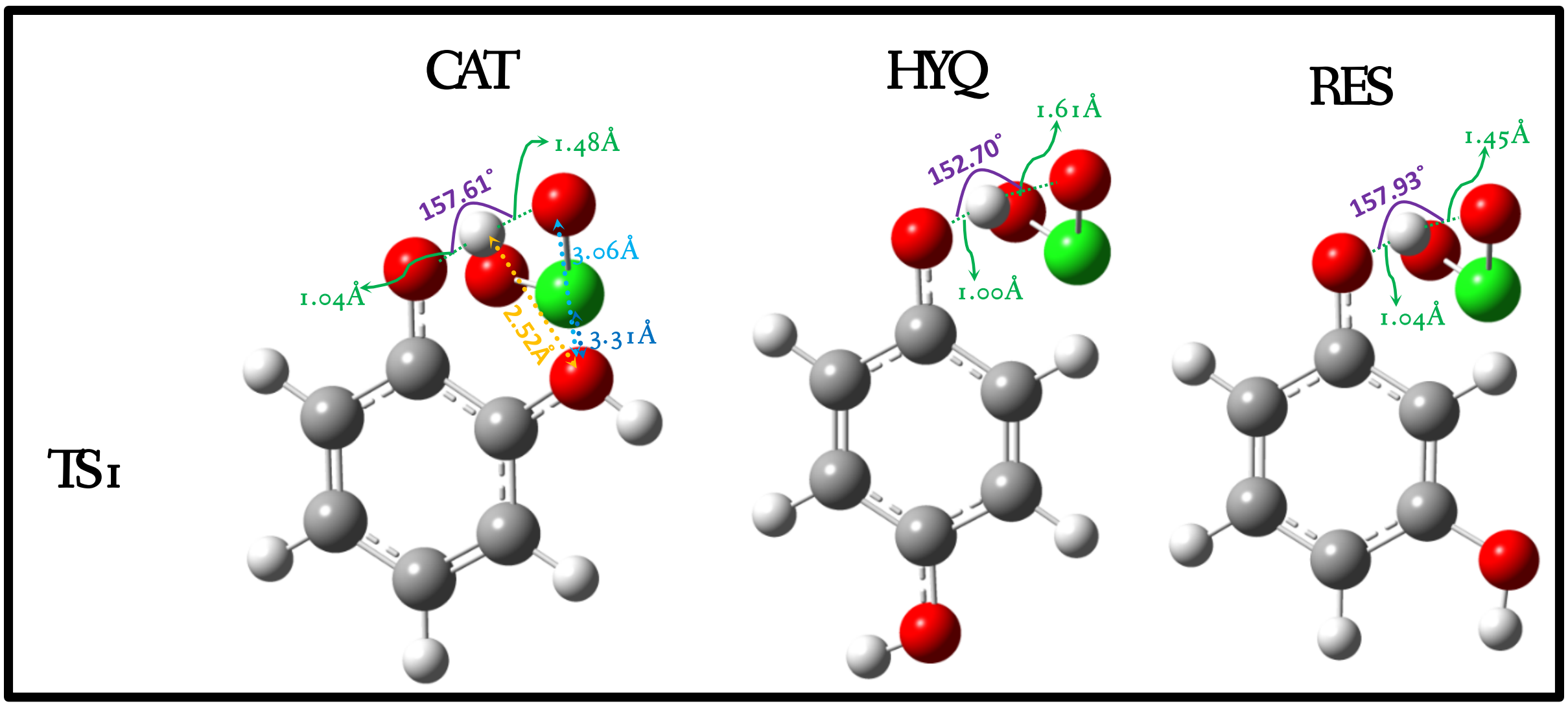

Figure S2: Optimized structures of TS2 and TS2' with selective bond lengths and bond angles calculated at the wB97XD/6-311+G(d,p) level of theory.

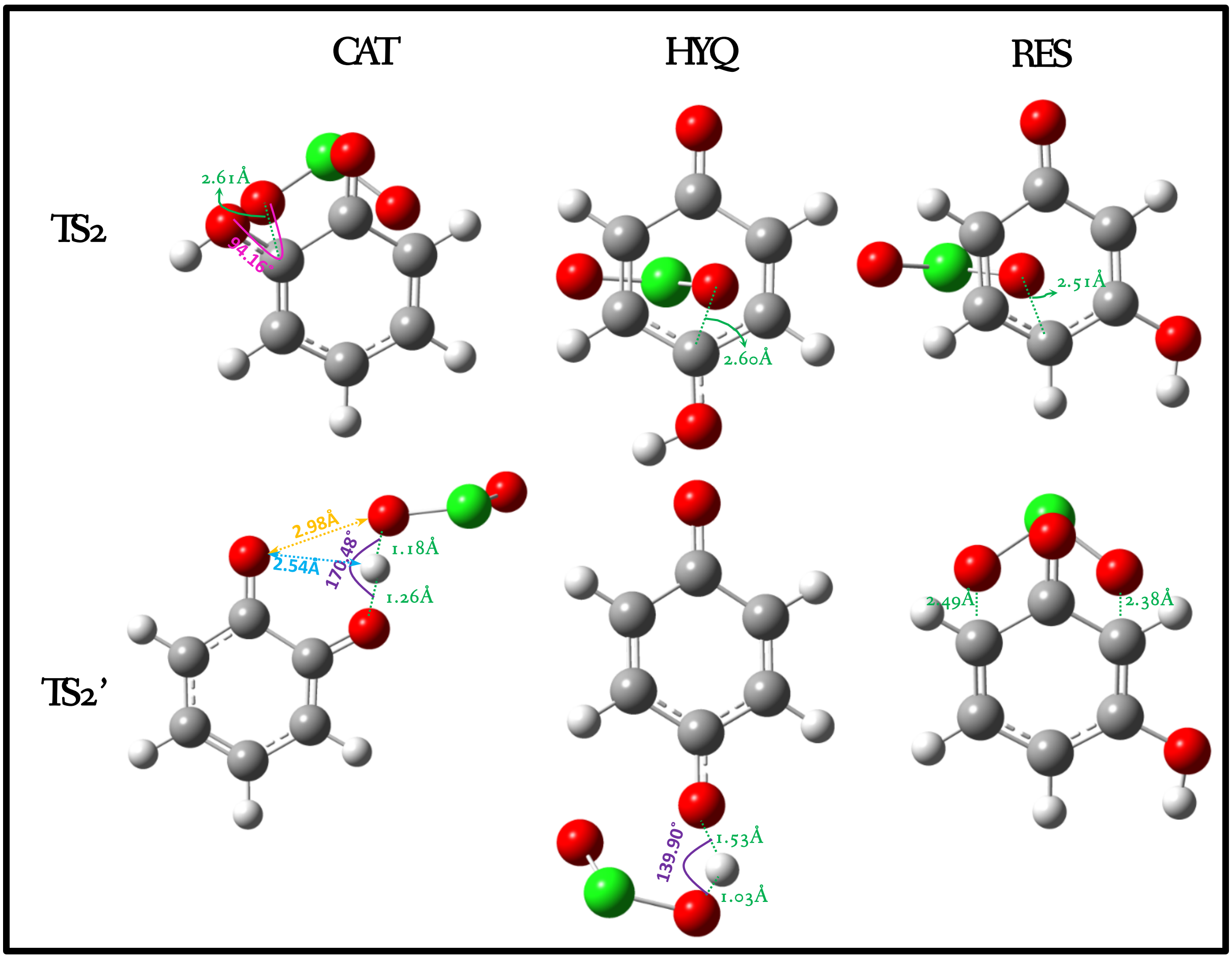

Figure S3: Optimized structures of TS3 and TS3' with selective bond lengths and bond angles calculated at the wB97XD/6-311+G(d,p) level of theory

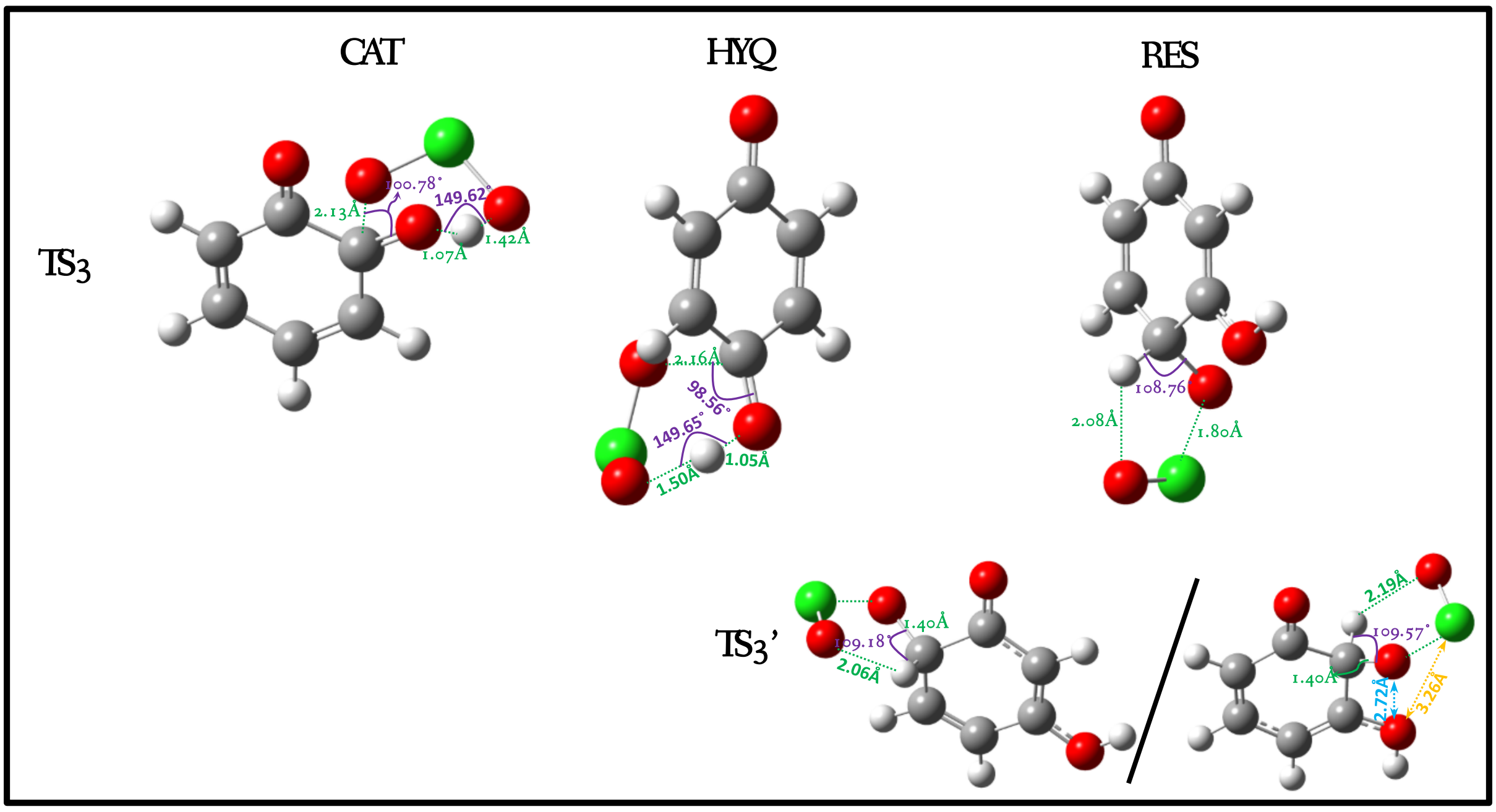

Figure S4: QTAIM parameters at the O...O interaction in CAT TS1

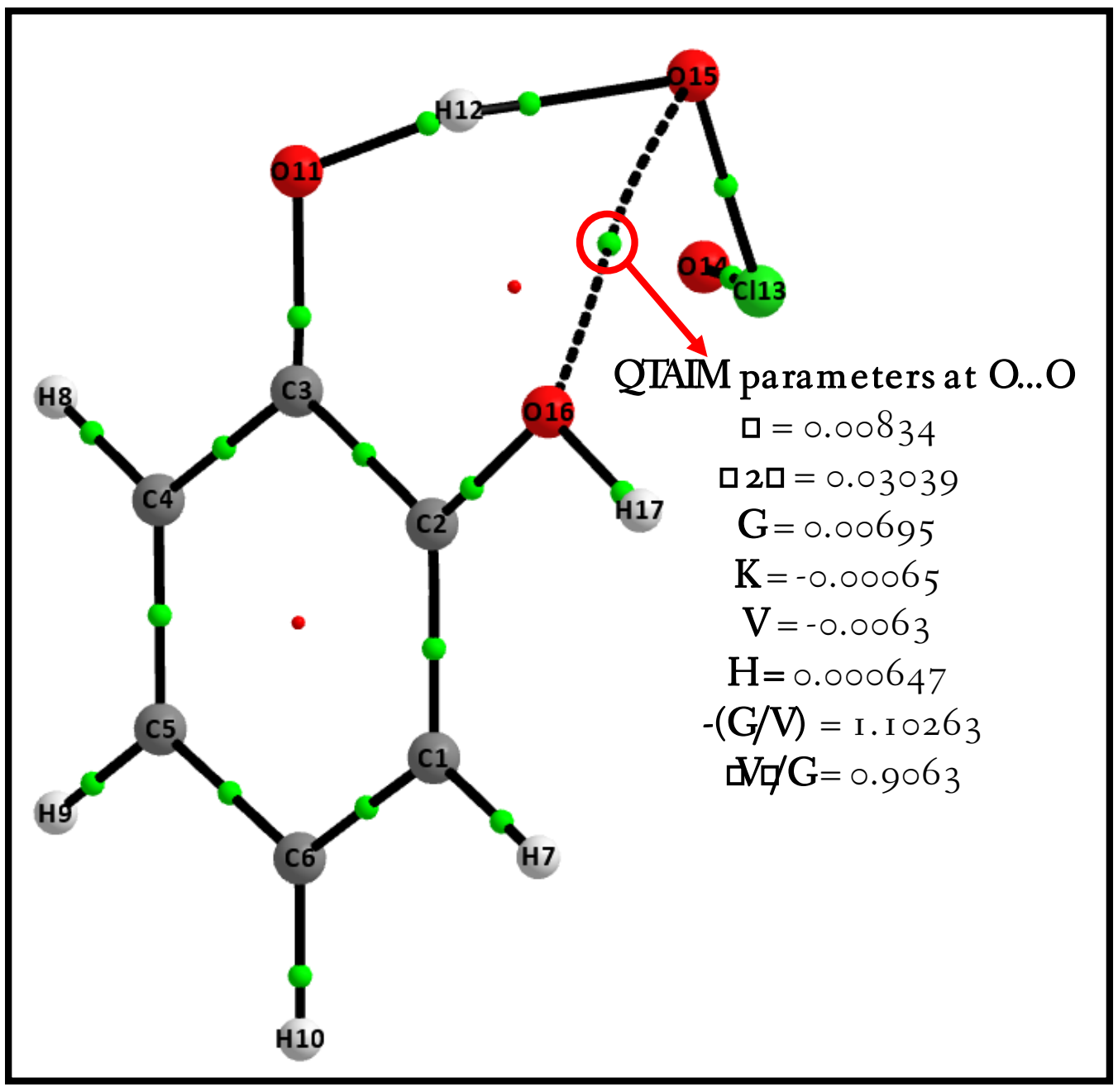

Figure S5: Free energy profile of the HAT/HAT pathway for RES calculated at the same level of theory, showing the energetically unstable meta-benzoquinone (meta-BQ) product..

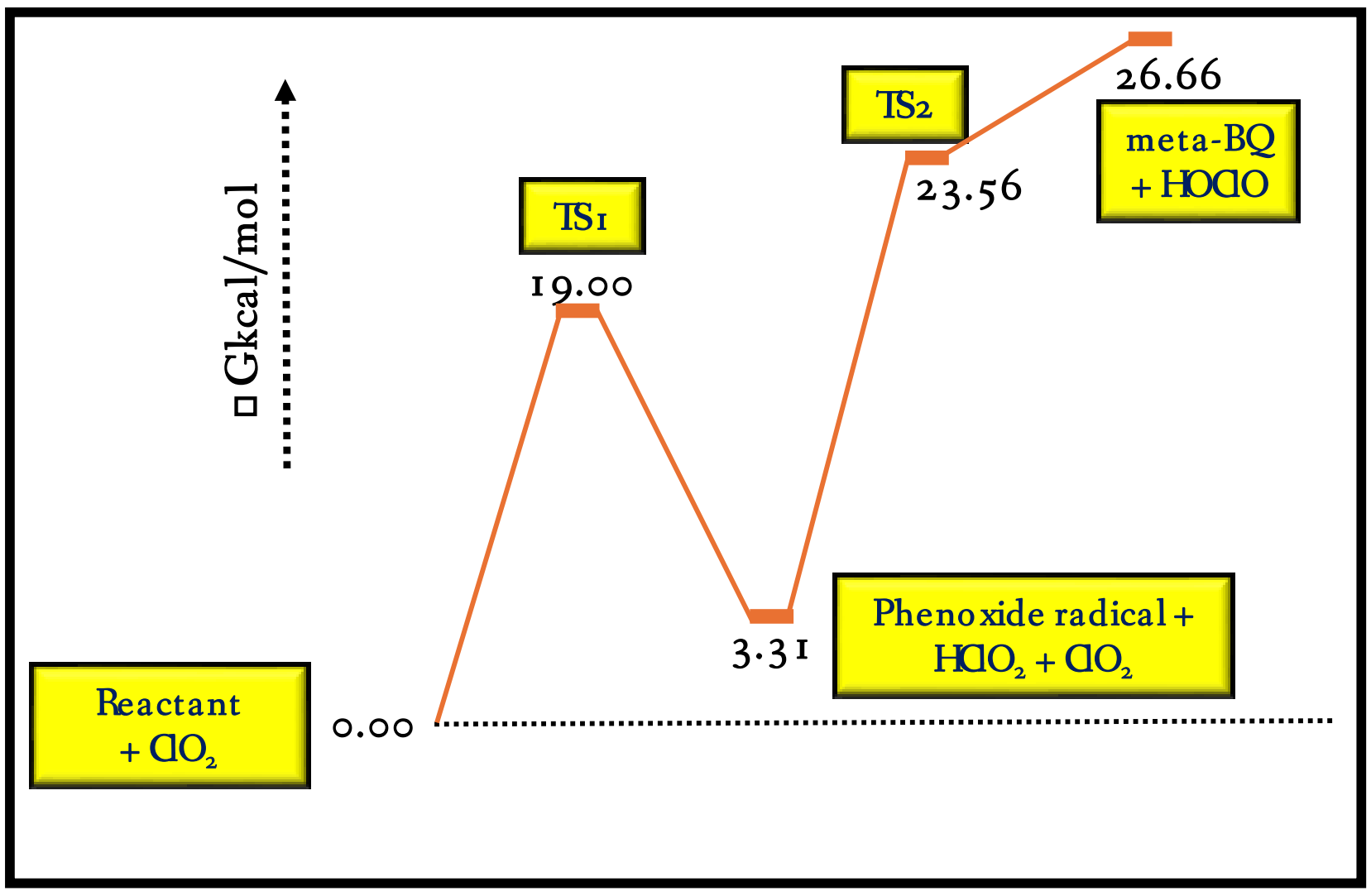

Figure S6: IRC graphs of all the transition state species identified in the present study

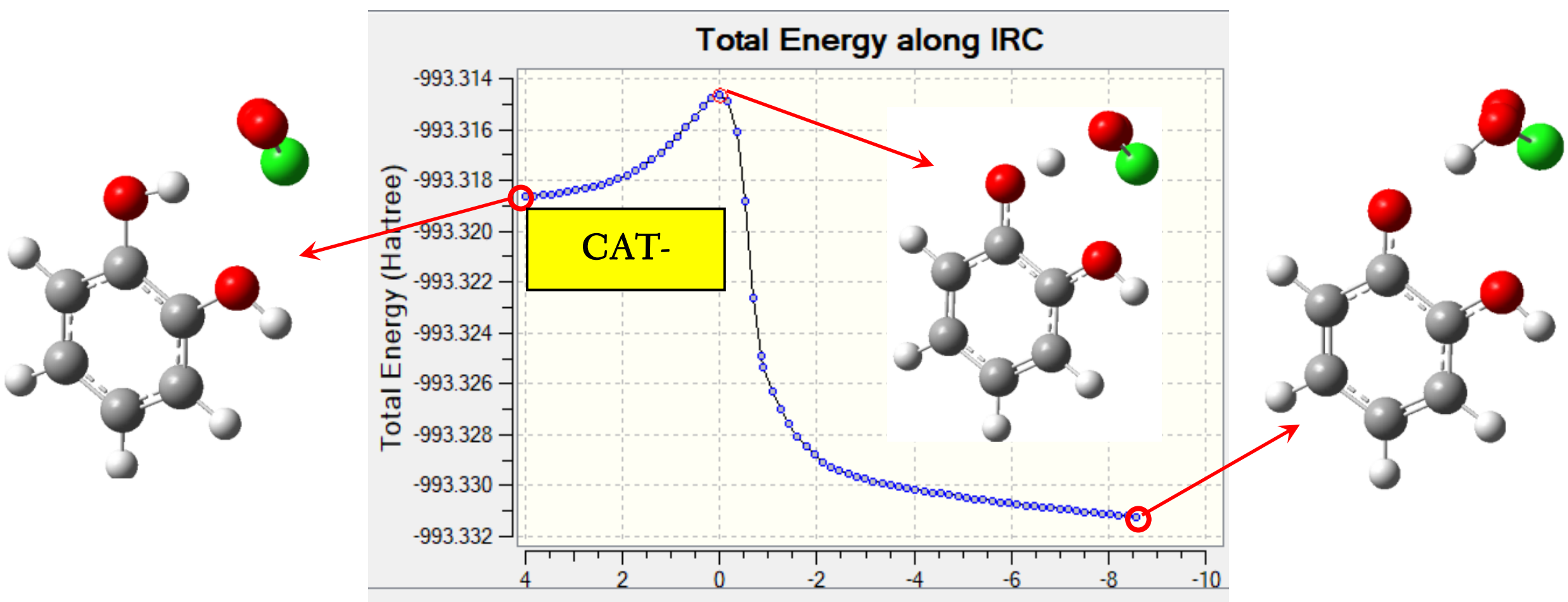

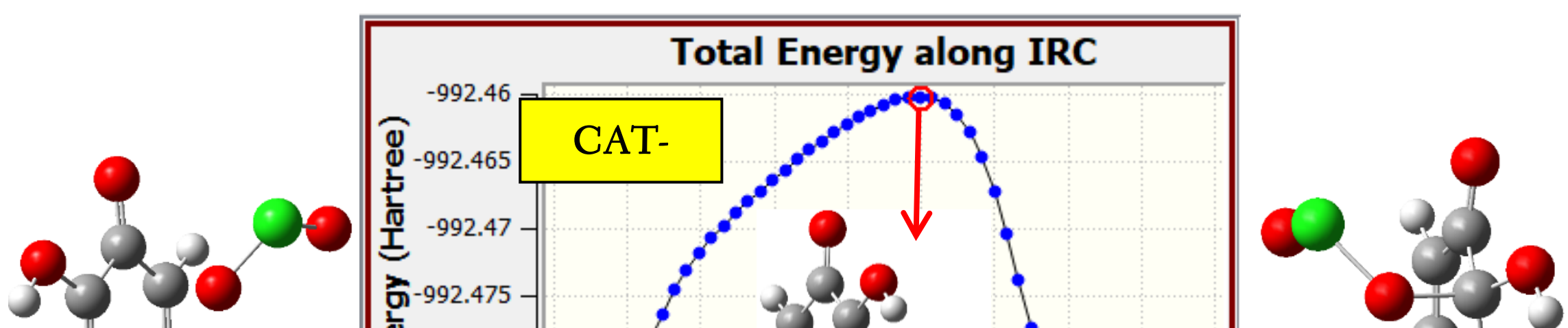

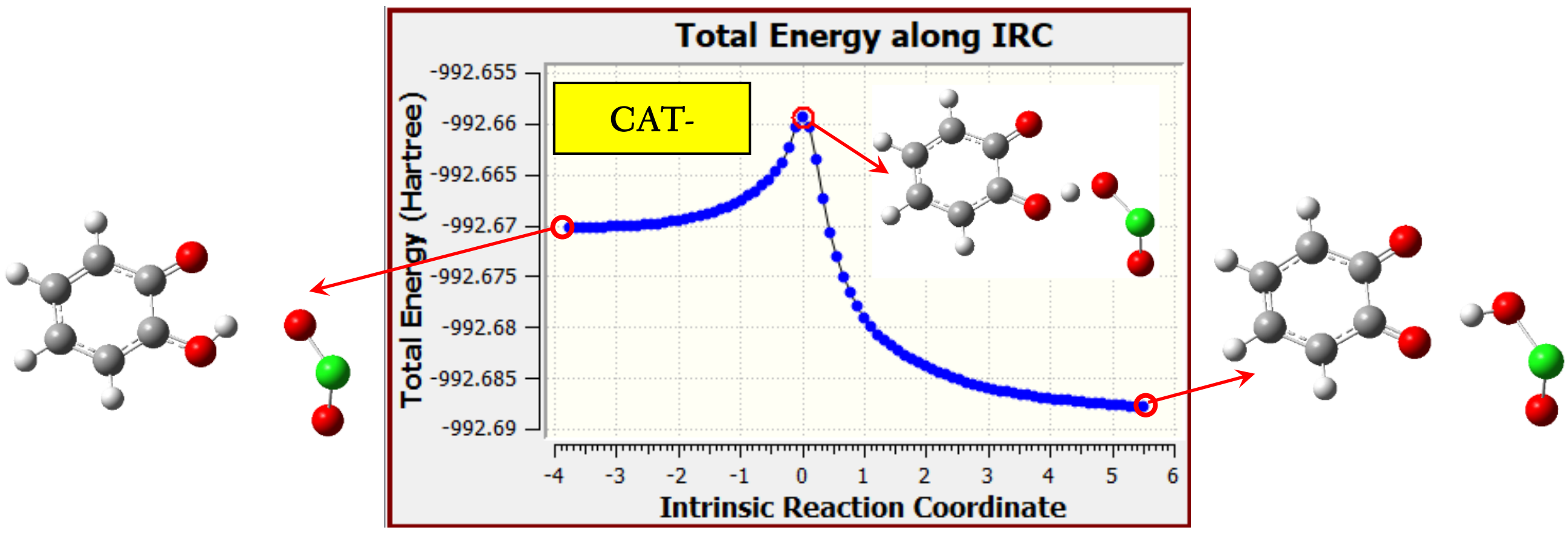
Total Energy along IRC
CAT-
Total Energy (Hartree)
-992.655
-992.66
-992.665
-992.67
-992.675
-992.68
-992.685
-992.69
-4 -3 -2 -1 0 1 2 3 4 5 6
Intrinsic Reaction Coordinate

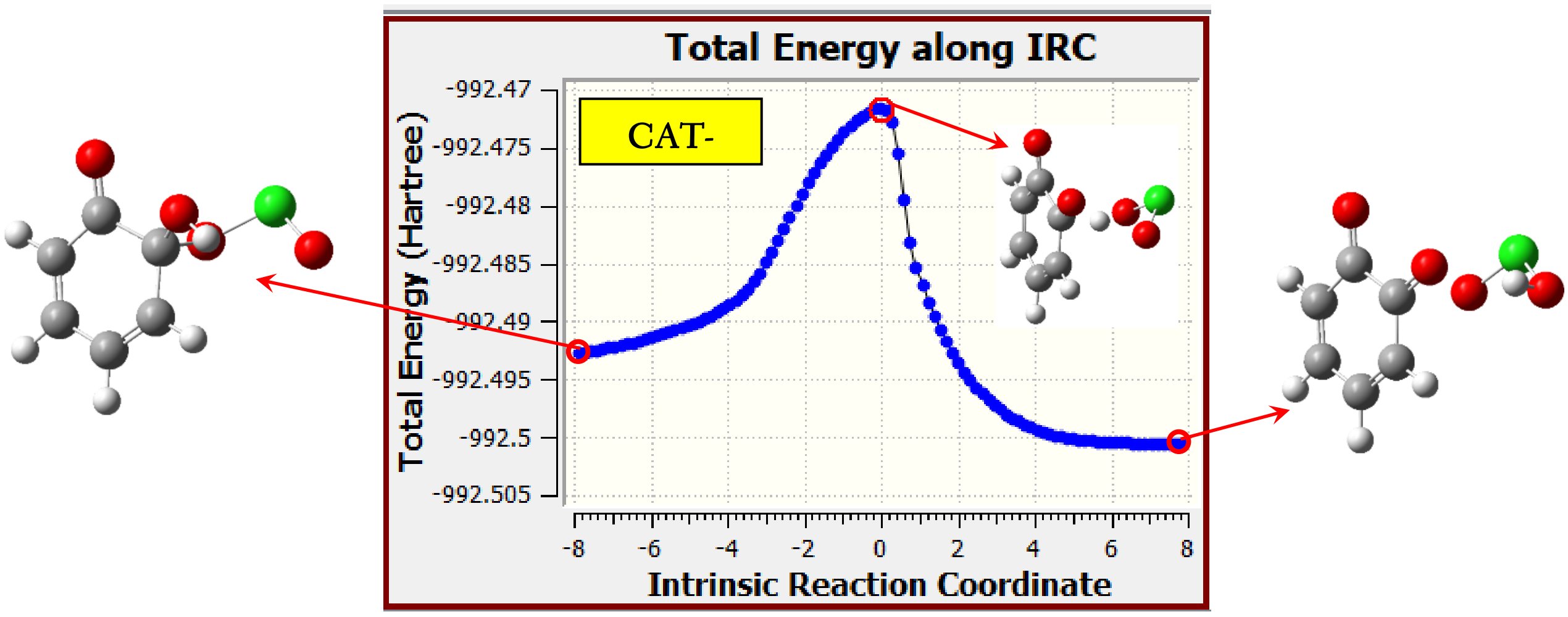
Total Energy along IRC
CAT-
Total Energy (Hartree)
-992.47
-992.475
-992.48
-992.485
-992.49
-992.495
-992.5
-992.505
-8 -6 -4 -2 0 2 4 6 8
Intrinsic Reaction Coordinate

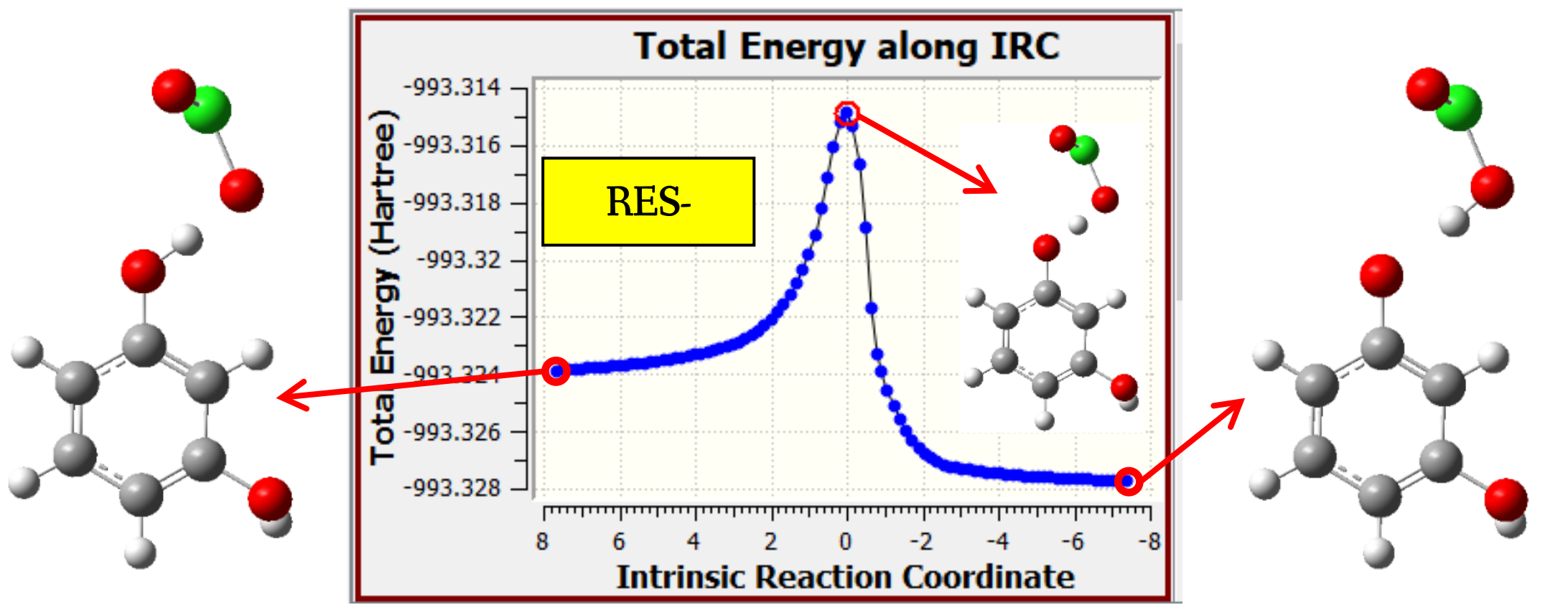
Total Energy along IRC
RES-
Total Energy (Hartree)
-993.314
-993.316
-993.318
-993.32
-993.322
-993.324
-993.326
-993.328
8 6 4 2 0 -2 -4 -6 -8
Intrinsic Reaction Coordinate

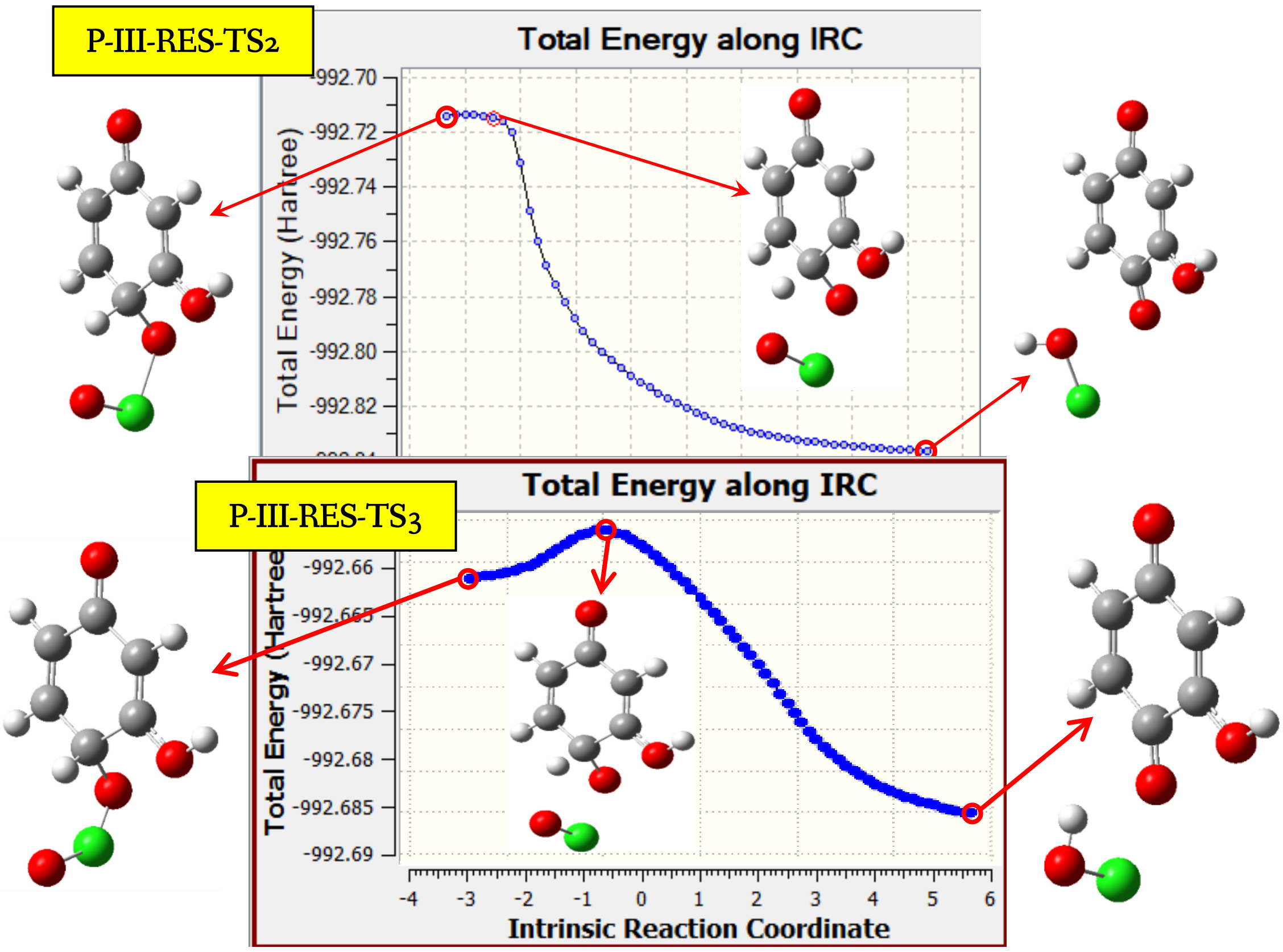
P-III-RES-TS2
Total Energy along IRC
Total Energy (Hartree)
-992.70
-992.72
-992.74
-992.76
-992.78
-992.80
-992.82
P-III-RES-TS3
Total Energy along IRC
Total Energy (Hartree)
-992.66
-992.665
-992.67
-992.675
-992.68
-992.685
-992.69
-4 -3 -2 -1 0 1 2 3 4 5 6
Intrinsic Reaction Coordinate

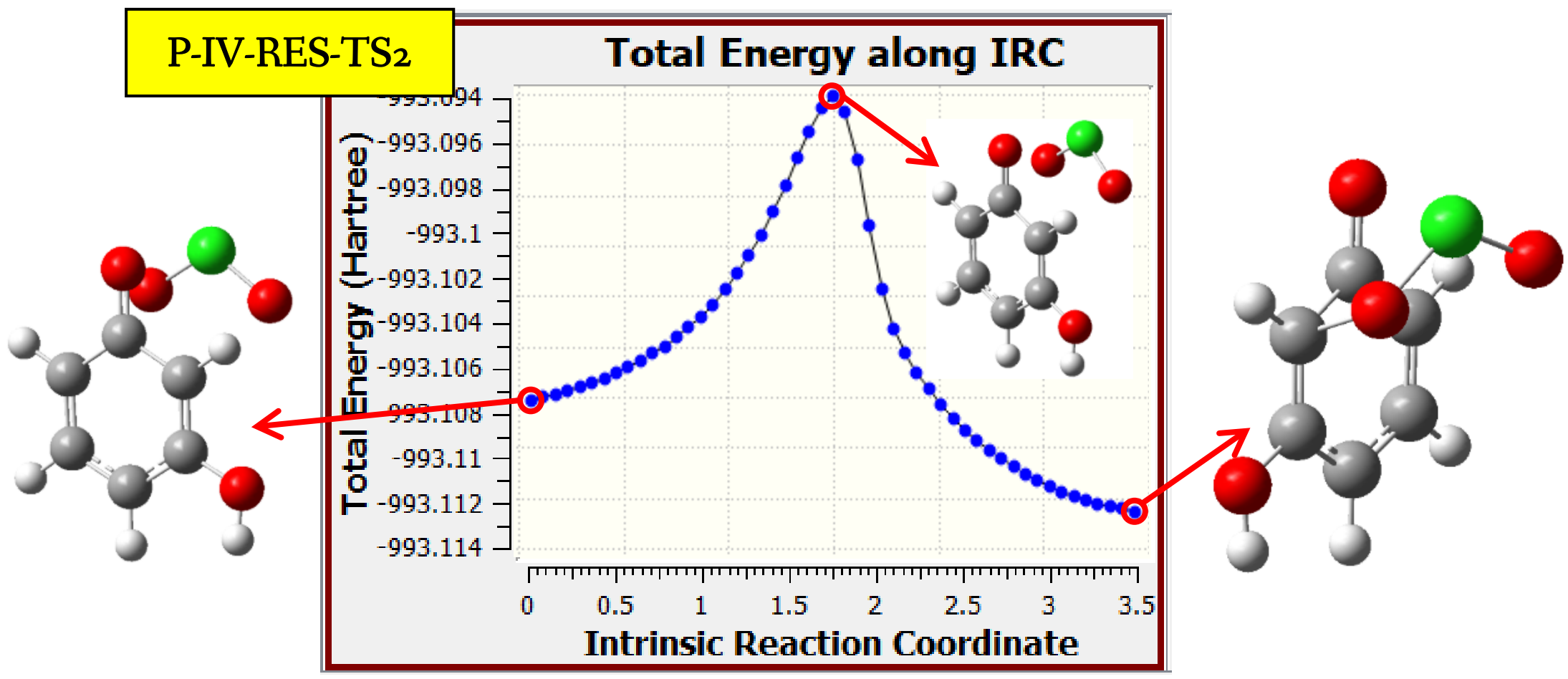
P-IV-RES-TS2
Total Energy along IRC
Total Energy (Hartree)
-993.096
-993.098
-993.1
-993.102
-993.104
-993.106
-993.11
-993.112
-993.114
0 0.5 1 1.5 2 2.5 3 3.5
Intrinsic Reaction Coordinate

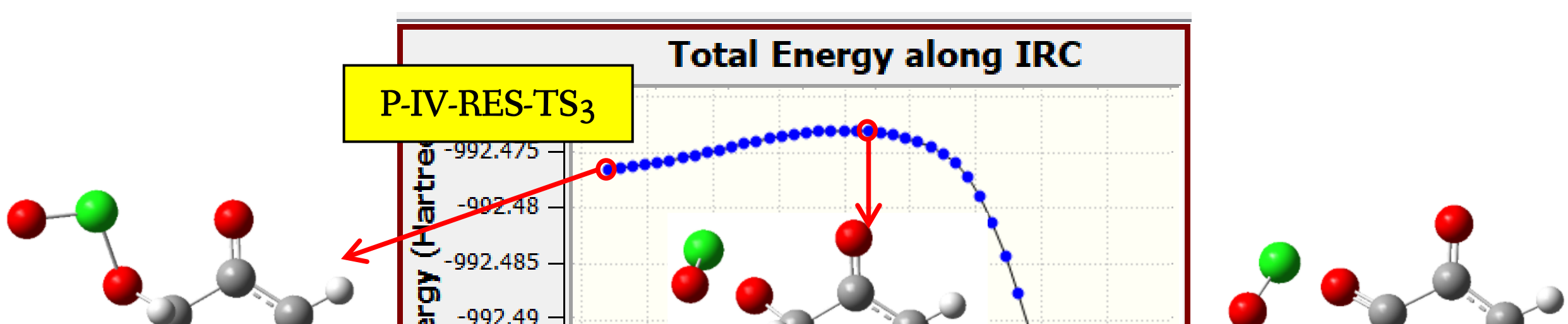
P-IV-RES-TS3
Total Energy along IRC
-992.475
-992.485

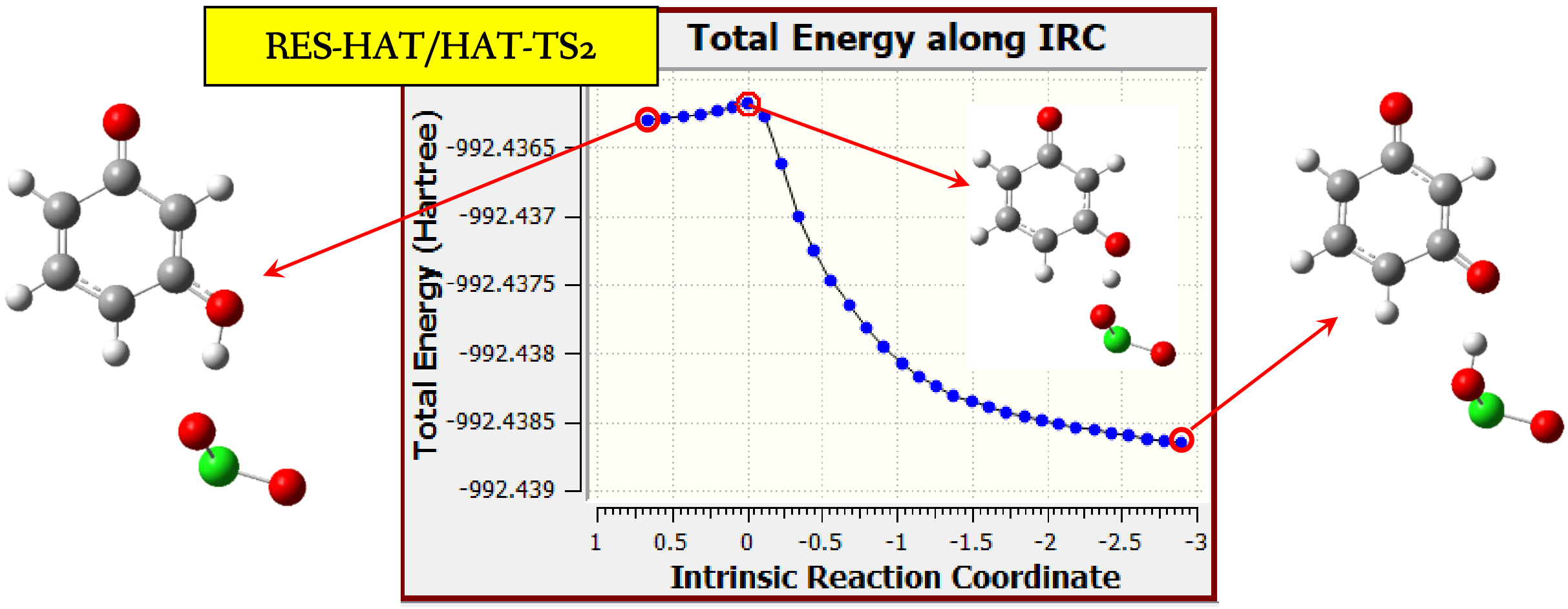
RES-HAT/HAT-TS2
Total Energy along IRC
Total Energy (Hartree)
-992.4365
-992.437
-992.4375
-992.438
-992.4385
-992.439
1
0.5
0
-0.5
-1
-1.5
-2
-2.5
-3
Intrinsic Reaction Coordinate

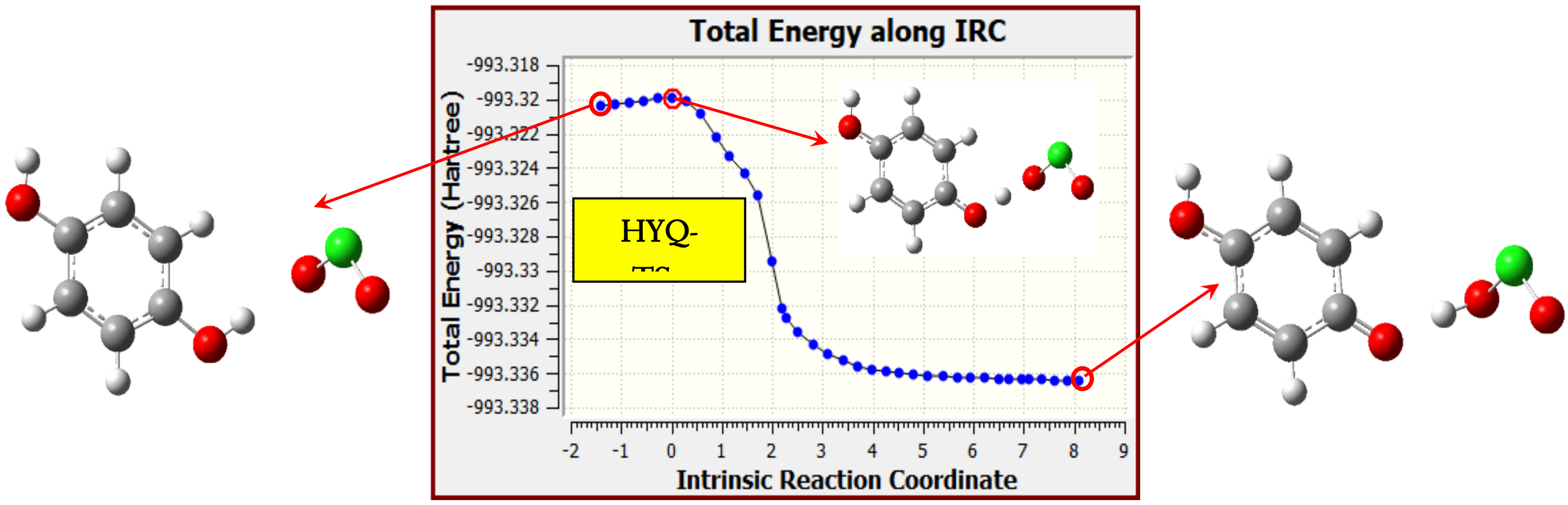
Total Energy along IRC
HYQ-
Total Energy (Hartree)
-993.318
-993.32
-993.322
-993.324
-993.326
-993.328
-993.33
-993.332
-993.334
-993.336
-993.338
-2
-1
0
1
2
3
4
5
6
7
8
9
Intrinsic Reaction Coordinate

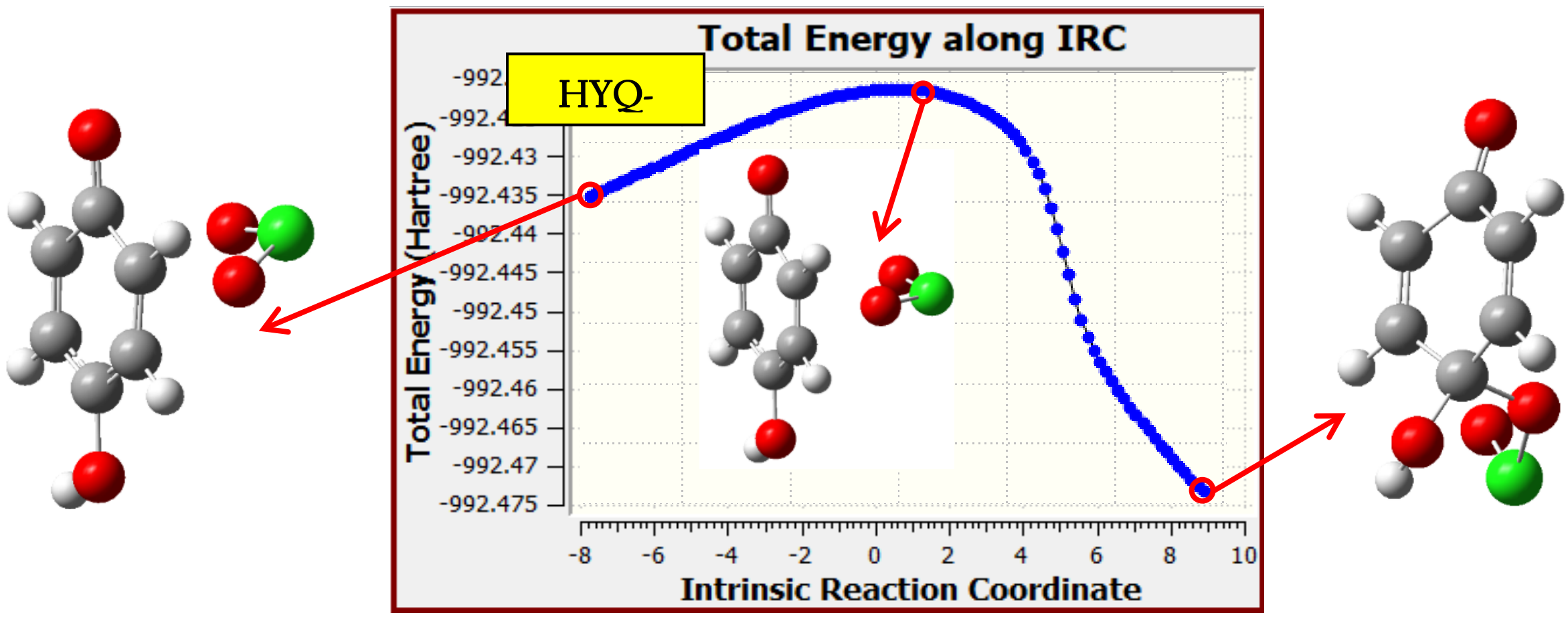

Total Energy along IRC
HYQ-
Total Energy (Hartree)
-992.43
-992.435
-992.445
-992.45
-992.455
-992.46
-992.465
-992.47
-992.475
-8 -6 -4 -2 0 2 4 6 8 10
Intrinsic Reaction Coordinate

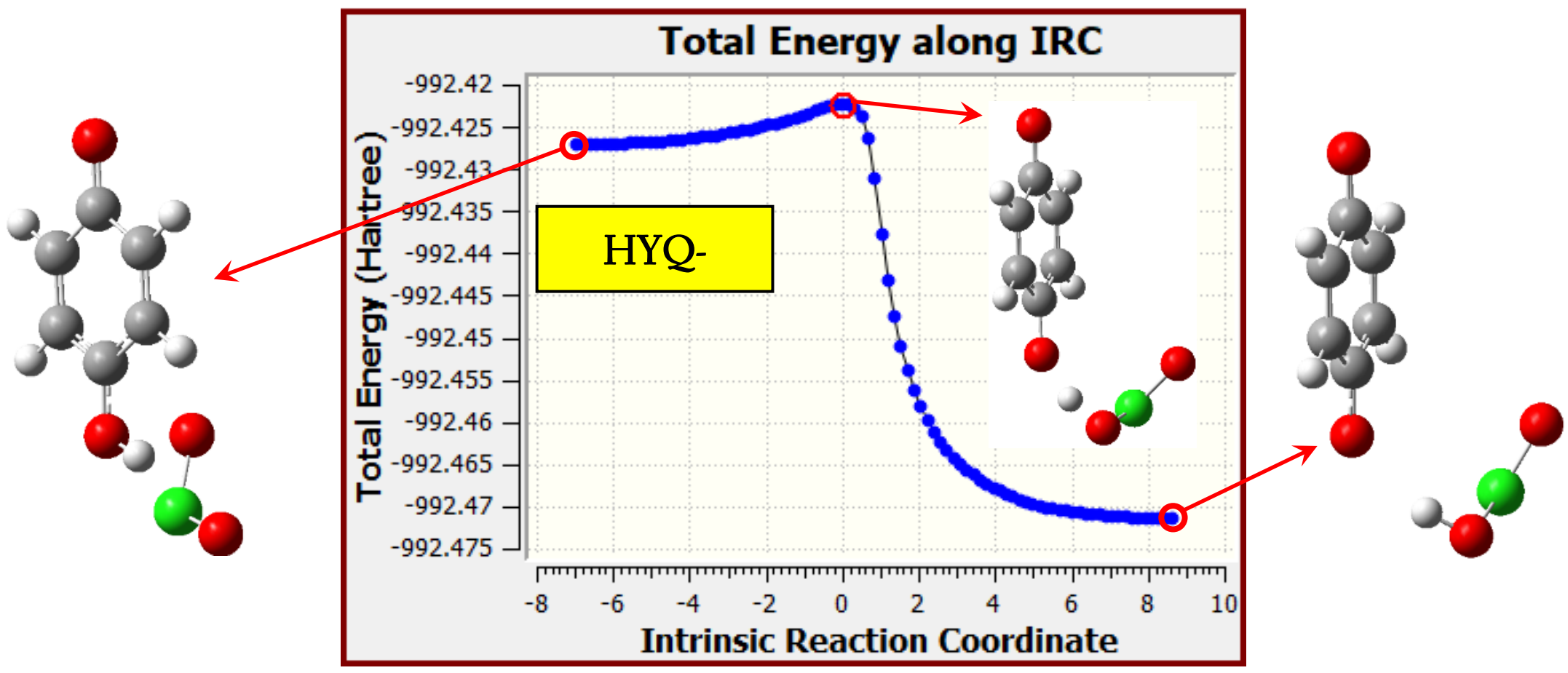

Total Energy along IRC
HYQ-
Total Energy (Hartree)
-992.42
-992.425
-992.435
-992.44
-992.445
-992.45
-992.455
-992.46
-992.465
-992.47
-992.475
-8 -6 -4 -2 0 2 4 6 8 10
Intrinsic Reaction Coordinate

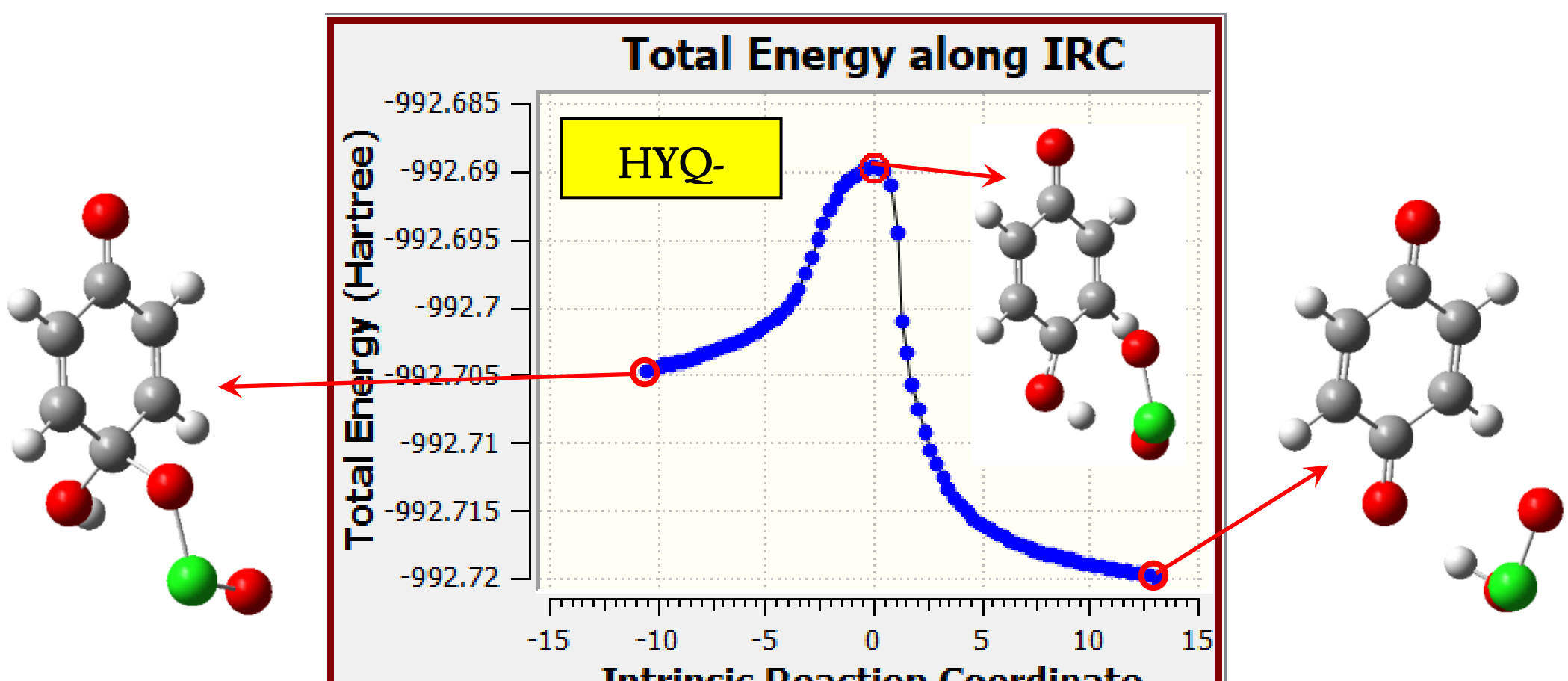

Total Energy along IRC
HYQ-
Total Energy (Hartree)
-992.685
-992.69
-992.695
-992.7
-992.71
-992.715
-992.72
-15 -10 -5 0 5 10 15

Table S1: Electronic energies with Gibbs free energy (EE + G) and enthalpy (EE + H) corrections for ClO□ and relevant reaction by-products calculated at the wB97XD/6-311+G(d,p) level of theory (in Hartree)

	EE+G	EE+H
ClO_2	-610.4465	-610.4167
$HClO_2$	-611.0758	-611.0454
Cl_2O	-995.5157	-995.4854
BrOCl	-3109.4995	-3109.4672
HOCl	-535.9759	-535.9491

Table S2: Electronic energies with Gibbs free energy (EE + G) and enthalpy (EE + H) corrections for all the reacting sp wB97XD/6-311+G(d,p) level of theory (in Hartree)

CA

	STEP - 1							STEP - 2								STEP - 3						
	R+2ClO2	TS1+ClO2	int1+ClO2+HClO	□G	kcal/mol	ΔG‡	kcal/mol	j/mol	int1+ClO2+HClO	TS2+HClO2	int2+HClO2	ΔG	kcal/mol	ΔG‡	kcal/mol	int2+HClO2	TS3+HClO2	pdt+2HClO	□G	kcal/mol	ΔG‡	kcal/mol
P1	-1603.495072	-1603.471051	-1603.49518	-0.00011	-0.06589	0.024021	15.07318	63066.17466	-1603.49518	-1603.467543	-1603.506	-0.010927	-6.85669	0.027634	17.340335	-1603.505999	-1603.481904	-1603.52	-0.02554	-16.0251	0.024095	15.11961
									int1+ClO2+HClO	TS2+HClO2	int2+2HClO2	P+2HClO2	□G(int2-R)	kcal/mol	ΔG(P-R)	kcal/mol	ΔG‡	kcal/mol				
P2									-1603.49518	-1603.472391	-1603.46486	-1603.52061	0.030317	19.02392	-0.025538	-16.025095	0.022786	14.29822				
	same step 1																		no step 3			

RES

	STEP - 1							STEP - 2								STEP - 3						
	R+2ClO2	TS1+ClO2	int1+ClO2+HClO	□G	kcal/mol	ΔG‡	kcal/mol	int1+ClO2+HClO2	TS2+HClO2	int2+HClO2	ΔG	kcal/mol	ΔG‡	kcal/mol	j/mol	int2+HClO2	TS3+HClO2	pdt+HClO2+H	□G	kcal/mol	ΔG‡	kcal/mol
P-III	-1603.49675	-1603.46647	-1603.49149	0.005267	3.305043	0.030283	19.0026	-1603.491486	-1603.44717	-1603.51615	-0.01939	-12.16848	0.044316	27.80829	116349.9	-1603.51615	-1603.51314	-1603.65712	-0.16036	-100.629	-0.01639	-10.2847
P-IV								-1603.491486	-1603.4573	-1603.51618	-0.01942	-12.1873	0.034188	21.45297	89759.23	-1603.51618	-1603.51411	-1603.65202	-0.15527	-97.43004	-0.01735	-10.8903
ortho-CRA-2								-1603.491486	-1603.45776	-1603.5113	-0.01455	-9.130125	0.033722	21.16055	88535.76	-1603.5113	-1603.5088	-1603.64527	-0.14852	-93.19693	-0.01205	-7.55949
								int1+ClO2+HClO2	TS2+HClO2	P+2HClO2	ΔG	kcal/mol	ΔG‡	kcal/mol								
HAT/HAT								-1603.491486	-1603.45393	-1603.45427	0.042484	26.65871	0.037553	23.56451								

HY

	STEP - 1							STEP - 2								STEP - 3						
	R+2ClO2	TS1+ClO2	int1+ClO2+HClO2	ΔG	kcal/mol	ΔG‡	kcal/mol	j/mol	int1+ClO2+HClO	TS2+HClO2	int2+HClO2	ΔG	kcal/mol	ΔG‡	kcal/mol	int2+HClO2	TS3+HClO2	pdt+2HClO2	ΔG	kcal/mol	ΔG‡	kcal/mol
P1	-1603.49398	-1603.47425	-1603.498134	-0.00415	-2.60475	0.019733	12.38246	51808.20218	-1603.498134	-1603.471285	-1603.509682	-0.015699	-9.85112	0.026849	16.8477475	-1603.509682	-1603.488435	-1603.52811	-0.03413	-21.4147	0.021247	13.33249
									int1+ClO2+HClO	TS2+HClO2	int2+2HClO2	P+2HClO2	□G(int2-R)	kcal/mol	ΔG(P-R)	kcal/mol	ΔG‡	kcal/mol				
P2									-1603.498134	-1603.479514	-1603.52811	-1603.52811	-0.02998	-18.8099	-0.034127	-21.4146925	0.01862	11.68405				
	same step 1																		no step 3			

Structure	Charge	Multiplicity	No. of imaginary frequencies	Imaginary frequency (cm^{-1})	$\langle S^2 \rangle$ before annihilation	$\langle S^2 \rangle$ after annihilation
CAT- Reactant	0	1	0	-	-	-
CAT-TS1	0	2	1	-1024.31	0.7662	0.7502
CAT-PR	0	2	0	-	0.7875	0.7509
CAT-TS2	0	1	1	-195.17	-	-
CAT-CHA	0	1	0	-	-	-
CAT-TS2′	0	1	1	-944.37	-	-
CAT-TS3	0	1	1	-544.19	-	-
CAT-Product	0	1	0	-	-	-
HYQ-Reactant	0	1	0	-	-	-
HYQ-TS1	0	2	1	-4370.06	0.7677	0.7502
HYQ-PR	0	2	0	-	0.7846	0.7508
HYQ-TS2	0	1	1	-57.19	-	-
HYQ-CHA	0	1	0	-	-	-
HYQ-TS2′	0	1	1	-683.51	-	-
HYQ-TS3	0	1	1	-312.71	-	-
HYQ-Product	0	1	0	-	-	-
RES-Reactant	0	1	0	-	-	-
RES-TS1	0	2	1	-1196.46	0.7577	0.7501
RES-PR	0	2	0	-	0.8018	0.7518
RES-TS2(P-III)	0	1	1	-134.20	-	-
RES-TS2(P-IV)	0	1	1	-170.51	-	-
RES-TS3(P-III)	0	1	1	-60.94	-	-
RES-TS3(P-IV)	0	1	1	-42.35	-	-
RES-CHA(P-III)	0	1	0	-	-	-
RES-CHA(P-IV)	0	1	01 1	-	-	-
RES-product(P-III)	0	1	0	-	-	-

RES-product(P-IV)	0	1	0	-	-	-
RES-HAT/HAT/TS2	0	1	1	-516.95	-	-
RES-meta-BQ	0	1	0	-	-	-

Table S3. Charge, spin multiplicity, number of imaginary frequencies, imaginary frequency values, and □S^2□ values for the optimized structures considered in the reaction pathways at wB97XD/6-311g+(d,p) level of theory with IEFPCM solvent model (solvent=water).

Table S4: Condensed Fukui Function values of phenoxide radical of RES

Atom	*f*	*f^+*	*f^0*
C1	*-0.04*	*-0.04*	*-0.04*
C2	*-0.09*	*-0.10*	*-0.10*
C3	*-0.08*	*-0.09*	*-0.08*
C4	*-0.13*	*-0.13*	*-0.13*
C5	*-0.05*	*-0.06*	*-0.06*
C6	*-0.15*	*-0.17*	*-0.16*

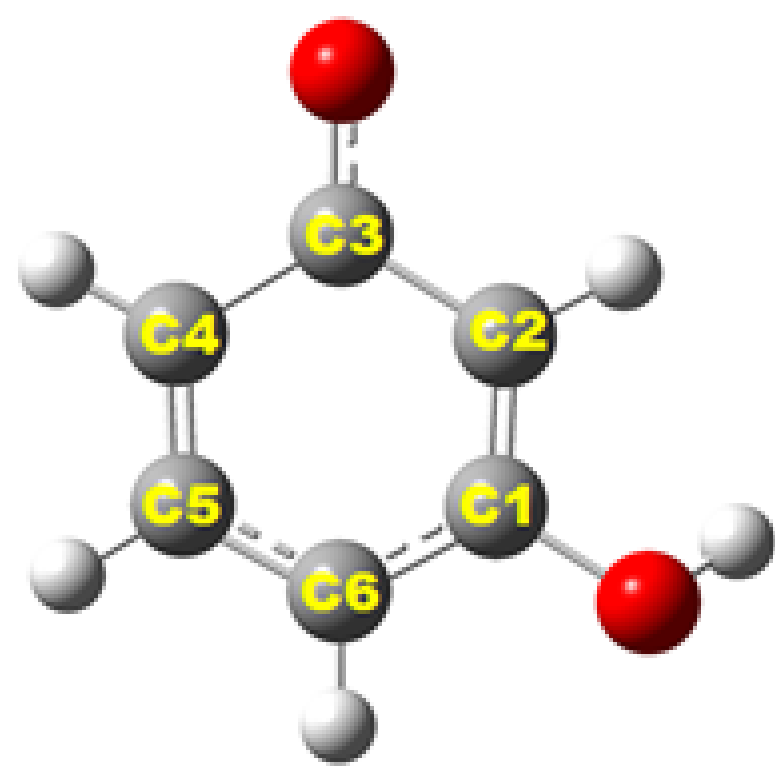

Table S5: Excited state properties calculated at the same level of theory for all the reactants, intermediates and products included in the reaction

Species	Parameter	Excited State 1	Excited State 2	Excited State 3	Excited State 4	Excited State 5
CAT	TV (eV)	5.0765	5.8224	5.8420	6.2844	6.5025
	λ (nm)	244.23	212.94	212.23	167.29	190.27
	f	0.1235	0.0731	0.0004	0.7390	0.8795
CAT-phenoxideRad	TV (eV)	1.7726	2.1409	2.8530	4.3340	4.5684
	λ (nm)	699.45	579.11	321.79	286.07	271.40
	f	0.000	0.021	0.0381	0.0393	0.1775

CAT-cyclohexadiene analogue	TV (eV)	3.0411	3.4934	3.8310	4.8578	5.0030
	λ (nm)	407.69	354.91	323.63	255.23	2247.82
	f	0.0004	0.0148	0.1249	0.0266	0.0053
O-BQ	TV (eV)	2.2232	3.0892	3.6569	5.7458	5.9147
	λ (nm)	557.69	401.35	339.04	215.78	209.62
	f	0.0001	0.0838	0.0000	0.3397	0.0011
RES	TV (eV)	1.1532	1.7109	3.3875	4.4509	4.9151
	λ (nm)	241.97	212.32	201.63	200.28	192.10
	f	0.0985	0.0419	0.000	0.7025	252.25
RES-phenoxideRad	TV (eV)	1.1532	1.7109	3.3875	4.4509	4.9151
	λ (nm)	819.36	724.67	366.00	278.56	252.25
	f	0.000	0.0181	0.1083	0.0034	0.1236
RES- cyclohexadiene analogue @para	TV (eV)	3.1683	3.8444	4.3169	5.0624	5.1441
	λ (nm)	391.32	322.51	287.20	244.91	241.91
	f	0.0001	0.000	0.1410	0.0058	0.2252
Hydroxy-1,4-benzoquinone	TV (eV)	3.1335	3.3632	3.3853	4.8088	6.4839
	λ (nm)	395.67	368.65	366.24	257.83	191.22
	f	0.000	0.0001	0.0587	0.6922	0.0011
RES- cyclohexadiene analogue @ortho	TV (eV)	3.1420	3.7572	4.1533	4.6752	5.0937
	λ (nm)	394.61	329.99	298.52	265.19	243.41
	f	0.0005	0.0707	0.0878	0.0289	0.0162

HYQ	TV (eV)	4.6967	5.7645	5.8569	6.1736	6.4813
	λ (nm)	263.98	215.08	211.69	200.83	191.29
	f	0.1525	0.1609	0.000	0.0025	0.9203
HYQ-phenoxide rad	TV (eV)	1.8408	2.8063	3.4093	3.9973	4.3951
	λ (nm)	673.69	441.81	363.67	310.17	282.10
	f	0.000	0.0023	0.1567	0.1006	0.1342
HYQ- cyclohexadiene analogue	TV (eV)	3.2567	3.5303	4.7584	5.0709	5.1716
	λ (nm)	380.70	351.20	260.56	244.50	239.74
	f	0.0001	0.0001	0.0073	0.0008	0.3210
P-BQ	TV (eV)	2.9348	3.2349	4.0045	4.8921	6.3005
	λ (nm)	422.47	383.27	309.61	253.44	169.78
	f	0.000	0.000	0.000	0.8164	0.0021

Where, TV-vertical excitation energy (eV); λ-excitation wavelength (nm); f-oscillator strength (dimensionless)

Table S6: Topological parameters at the BCP for TS1, TS2, TS2' TS3 and TS3' computed at the same level of theory

	BCP	ATOMS	ρ	∇2ρ	G	K	V	-(G/V)
**CAT-TS1**	24	H12…O15	0.081252	0.150449	0.062809	0.025197	-0.088006	0.7137
**CAT-TS2**	30	C3….O11	0.047560	0.122852	0.032776	0.002063	-0.034839	0.9408
	19	C6…..O19	0.020460	0.061188	0.013995	-0.001302	-0.012693	1.1028
**CAT-TS3**	21	O1…C13	0.0349	0.1207	0.0349	0.0048	-0.0397	-0.0048

	32	H15…O7	0.0979	0.1242	0.0718	0.0407	-0.1125	-0.0407
**CAT-TS2’**	33	O16….O15	0.009483	0.031836	0.007372	-0.000587	-0.006785	1.0862
	26	O15….H12	0.027421	0.089887	0.021619	-0.000853	-0.020766	1.0410
	27	Cl13….C3	0.028696	0.067184	0.01700	0.000200	-0.017196	0.9884
**RES-TS1**	26	O14….H11	0.084437	0.180626	0.071599	0.026442	-0.098041	0.7303
	30	Cl12….C3	0.010163	0.035327	0.006937	-0.001895	-0.005042	1.3757
**RES-TS2**	23	O11….C6	0.024319	0.086558	0.019482	-0.002158	-0.017324	1.1246
	31	O13….C4	0.007872	0.023211	0.005033	-0.000769	-0.004264	1.1805
**RES-TS3**	23	O11….H8	0.024134	0.082423	0.018215	-0.002390	-0.015825	1.1510
**RES-TS2’**	18	O10…C6	0.026136	0.074771	0.017572	-0.001121	-0.016451	1.0684
	19	Cl11….O13	0.019336	0.075719	0.015910	-0.003020	-0.012890	1.2343
	31	O12….C4	0.032250	0.089003	0.021713	-0.000538	-0.021175	1.0254
**RES-TS3’**	21	O12….H9	0.025461	0.085682	0.019056	-0.002364	-0.016692	1.1410
**HYQ-TS1**	24	O13….H10	0.058346	0.150061	0.048016	0.010500	-0.058516	0.8205
	30	Cl11….C3	0.012189	0.039522	0.007793	-0.002088	-0.005704	1.3667
**HYQ-TS2**	23	O10….C6	0.020036	0.074648	0.016241	-0.002421	-0.013820	1.1751
	32	O10……C2	0.019583	0.087075	0.018321	-0.003448	-0.014873	1.2318
**HYQ-TS3**	19	O12….C6	0.048355	0.116209	0.032603	0.003551	-0.036154	0.9017
	34	O14…..H16	0.080910	0.145821	0.062526	0.026071	-0.088597	0.7058

HYQ-TS2'	21	O13....H14	0.124876	0.488796	0.084394	0.072174	-0.156569	0.5389
	34	O2....H16	0.015162	0.046149	0.010148	-0.001389	-0.008759	1.1589

Table S7: Coordinates of all the structures included in this study computed at wB97XD/6-311g+(d,p) level of theory with IEFPCM solvent model (solvent=water).

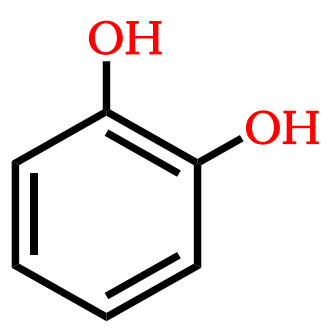

C -1.83110000 -1.27683800 0.01310600
C -2.36093900 0.00667600 -0.02206300
C -1.51494900 1.11330400 -0.01763600
C -0.14094200 0.93479800 0.02171800
C 0.39101000 -0.35872400 0.05713500
C -0.45045500 -1.46025200 0.05300800
H -3.43396700 0.15430000 -0.05427100
H -1.91120000 2.12192100 -0.04555300
O 0.68396600 2.01659300 0.02534500
H 1.59620700 1.70805200 0.05188300
H -2.48518900 -2.14063400 0.00967600
H -0.02539100 -2.45820100 0.08101700
O 1.75431800 -0.42568600 0.09439500
H 2.05384300 -1.33757600 0.10853600

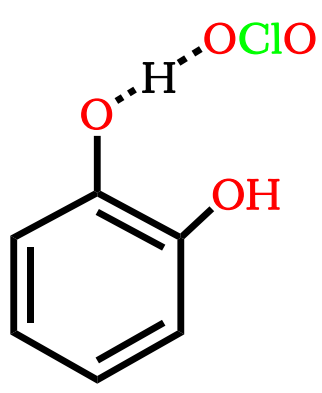

C -1.99512500 1.57292500 -0.28839500
C -1.06160600 0.79278100 -0.97966400
C -0.94670000 -0.61077200 -0.69567600
C -1.83120000 -1.19180500 0.23508300
C -2.75048300 -0.41395900 0.88807800
C -2.82604400 0.97559800 0.63098200
H -2.06651500 2.63426800 -0.49684900
H -1.73932800 -2.25359900 0.42561000
H -3.42067000 -0.85772300 1.61344900
H -3.55213900 1.57735800 1.16336400
O -0.05760100 -1.34998800 -1.31830200
H 0.87433000 -0.90041900 -1.28655100
Cl 1.60447700 0.39139500 0.67210400
O 1.70643800 -0.57415300 1.87352000
O 2.07761400 -0.27821200 -0.68080400
O -0.25492300 1.26150000 -1.92509500
H -0.35440900 2.21391600 -2.03186700

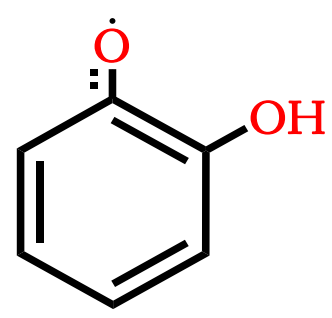

C -0.72312400 -1.43243600 0.07310800
C -0.05325600 -0.21712000 0.08569600
C -0.78970500 1.04845300 0.04144300
C -2.23145200 0.95060400 -0.01770300
C -2.86221100 -0.25848800 -0.02915300

C -2.10897700 -1.45613300 0.01649100
H -2.78296900 1.88310100 -0.05243800
H -3.94332500 -0.31311600 -0.07459700
H -2.62180300 -2.41039000 0.00702100
O -0.19198500 2.14053700 0.05495900
H -0.16204500 -2.36042800 0.10643300
O 1.27557100 -0.10595800 0.13760500
H 1.68751900 -0.97560700 0.16504600

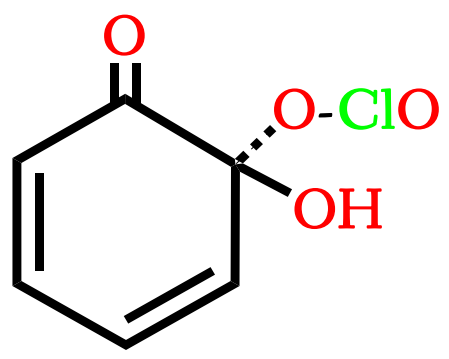

C 1.53875100 -1.28993800 -0.86487500
C 1.82513800 0.07587400 -0.98018200
C 1.47822500 1.03592400 -0.02769900
C 0.74670000 0.65860500 1.07381900
C 0.47259400 -0.78248800 1.32568600
C 0.87914800 -1.75200700 0.24726500
H 1.85987500 -1.97964400 -1.63598700
H 2.37853000 0.39675700 -1.85643200
O -1.59733500 -1.32630900 -0.46795700
Cl -2.24862800 -0.27012700 0.47845000
O -1.30541100 0.99777000 0.46011300
O -0.06587000 -1.16307600 2.34211700
H 1.75921800 2.06997800 -0.17367200
O 0.67536800 -3.01485900 0.54974600
H 0.97882800 -3.59568200 -0.15754900
H 0.56340100 1.32132700 1.90802300

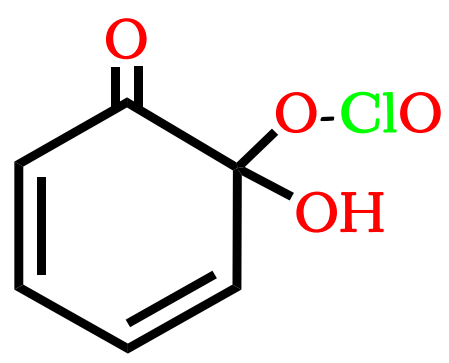

C 0.05178300 -0.33401700 -0.01063500
C 1.25245700 0.23865200 -0.08760700
C 1.43071200 1.67920100 0.09672000
C 0.39867100 2.51929600 0.27426500
C -0.96958400 2.01163700 0.23757200
C -1.16346800 0.47500700 0.35024200
O -2.15970200 0.04950800 -0.58632800
Cl -3.78300800 0.49431400 -0.16700300
O -4.51828200 -0.77554800 0.28874500
H -0.09806200 -1.40070900 -0.13623800
O -1.95030000 2.71610900 0.13053600
H 2.13117700 -0.36256200 -0.28668100
H 2.44137200 2.07036300 0.06402100
H 0.53121800 3.59028800 0.36494000
O -1.55739900 0.27148500 1.66698700
H -1.43552900 -0.65387100 1.90665800

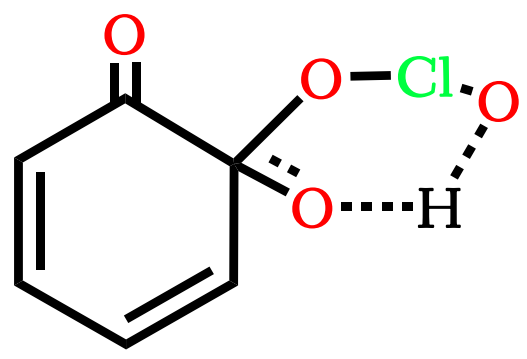

C -0.20620400 0.29450300 0.22978600
C 0.11260100 0.07102800 1.64277800
C 1.39032400 -0.02932000 2.03702900
C 2.50658600 0.03381600 1.09478600
C 2.32388800 0.12608900 -0.23120800
C 0.97309700 0.20266400 -0.75151000
H -0.71519400 0.05422100 2.34055200
H 3.51111400 0.00124400 1.49887100
H 3.14524200 0.15321300 -0.93582000
O 0.81892200 -1.92132000 -0.84099100
Cl 0.68656000 -2.18315600 -2.41662200
O 1.82865900 -1.27919600 -3.13643400
O 0.71492400 0.51153300 -1.95986500
H 1.36647600 -0.03675400 -2.62165200
O -1.31212000 0.53717300 -0.19004100
H 1.62561700 -0.15172700 3.08764200

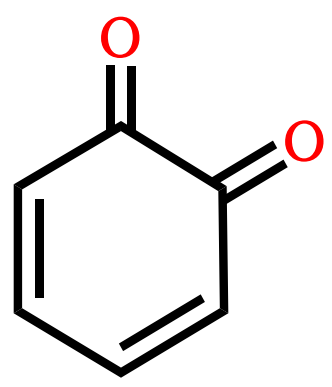

C -1.70478900 0.14709300 0.00086100
C -0.23908000 0.15165700 0.00096200
C 0.47921300 1.28397900 0.00072700
C -0.19831800 2.58471500 0.00153000
C -1.76056100 2.57982300 -0.00424200
C -2.43005500 1.27496600 -0.00135300
H -3.51357500 1.26822900 -0.00219600
O 0.38659900 3.64371100 0.00415000
O -2.35210000 3.63509400 -0.01087200
H 1.56275300 1.28391000 0.00112100
H -2.20097100 -0.81663600 0.00222400
H 0.26293500 -0.80905400 0.00137000

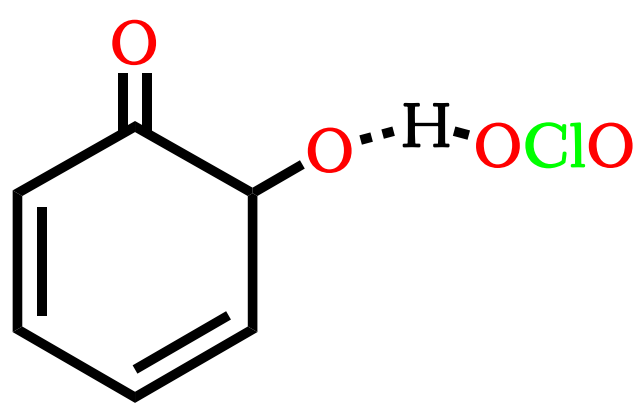

C -1.89986100 1.58698900 -0.28169000
C -0.98525900 0.76734500 -1.07666800
C -0.84809700 -0.67516500 -0.65625300
C -1.72627400 -1.24266000 0.29437800
C -2.60656400 -0.41929900 0.91926200
C -2.68215600 1.00264600 0.64419800
H -1.95307700 2.64370100 -0.51056500
H -1.65061900 -2.29725200 0.52284400
H -3.27784500 -0.82758900 1.66509200
H -3.39113300 1.59242900 1.21150300
O 0.00984800 -1.40445400 -1.27944400

H 0.68998500 -0.83151800 -1.70242600
Cl 1.15660300 0.37512800 0.78778600
O 1.22565900 -0.73160900 1.85873800
O 2.00464000 0.06125300 -0.47744400
O -0.34775100 1.15059800 -2.03873000

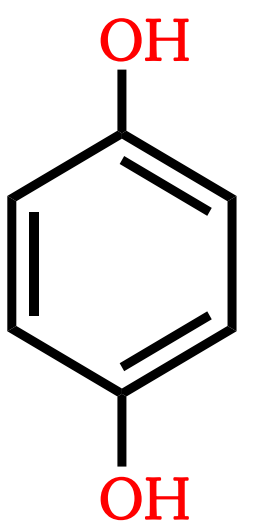

C -1.74473000 -1.34321300 0.14578800
C -2.31776800 -0.07459000 0.14174600
C -1.51385600 1.05629300 0.06105800
C -0.13179100 0.92724900 -0.01679900
C 0.44140500 -0.34138400 -0.01325200
C -0.36249900 -1.47211200 0.06740000
H -3.39553100 0.03735300 0.20250500
H -1.95910800 2.04431000 0.05815100
O 0.61011000 2.07288600 -0.09499300
H 1.54453600 1.85630800 -0.13648100
H 1.51914600 -0.45332200 -0.07460900
H 0.08259500 -2.46020500 0.06997000
O -2.48651700 -2.48863900 0.22373400
H -3.42073600 -2.27219300 0.26971700

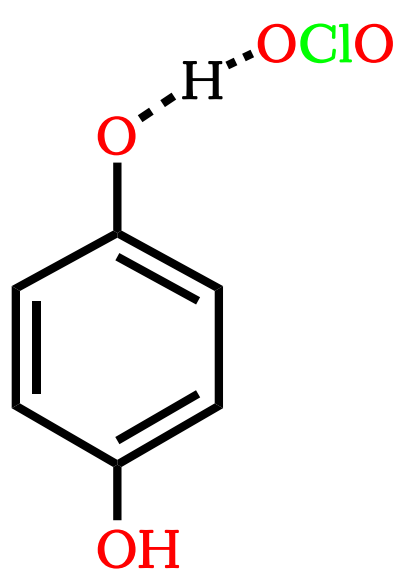

C -1.99959600 1.53291500 -0.38229500
C -1.02621600 0.73826700 -0.93923800
C -0.97606400 -0.64508700 -0.63890900
C -1.93894900 -1.20198800 0.22943100
C -2.91250600 -0.40728600 0.77919100
C -2.94655600 0.97040200 0.48368800
H -1.89038900 -2.26102700 0.44949200
H -3.65231100 -0.83383800 1.44752500
O -0.07449000 -1.45220900 -1.18187800
H 0.80000600 -0.97333200 -1.28308500
Cl 1.64097700 0.43388300 0.69128800
O 1.95588300 -0.52618400 1.84876900
O 2.05615100 -0.14657000 -0.70217000
H -0.30154200 1.15689600 -1.62754600
O -3.86593900 1.79185000 1.00437600
H -4.46627100 1.31774200 1.58827900
H -2.04912700 2.59196000 -0.60118200

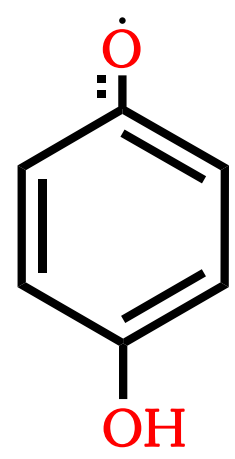

C -0.80894200 -1.42619000 0.14144300
C -0.11435300 -0.25404900 0.04243900
C -0.79353900 1.02247900 -0.05365000
C -2.24334100 0.98904700 -0.03699500
C -2.92596000 -0.18723400 0.06244300
C -2.22046900 -1.40779900 0.15130100
H -2.76770800 1.93494000 -0.10795400
H -4.00925000 -0.21405200 0.07456400
O -0.16155700 2.09663900 -0.14695300
H 0.96948300 -0.24907400 0.03218700
H -0.28640900 -2.37437000 0.21293800
O -2.95216900 -2.52376500 0.24258400
H -2.39226400 -3.30524400 0.29088900

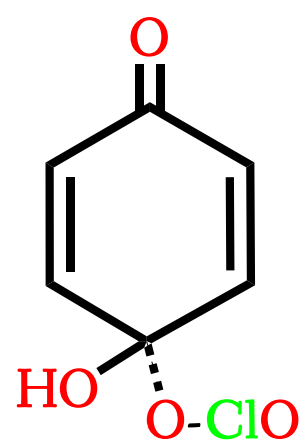

C 1.57451900 -1.37464200 -0.87813600
C 1.77955000 -0.07197700 -1.12713200
C 1.46925700 0.95988400 -0.10831600
C 1.01432400 0.48463700 1.22312200
C 0.79730900 -0.81694500 1.45082700
C 0.96645900 -1.74924900 0.36556800
H 0.87144500 1.23498500 1.99057600
H 0.45030000 -1.18490200 2.40933000
O 1.63392900 2.14389700 -0.33152900
O -0.80346200 -0.29572300 -0.85633400
Cl -2.27622200 0.17472800 -0.47526100
O -2.39063100 0.53048100 1.05496800
H 2.18529100 0.27704700 -2.06875500
H 1.77551600 -2.15214100 -1.60337500
O 0.66783200 -3.00282000 0.49896400
H 0.26462600 -3.20445800 1.35471100

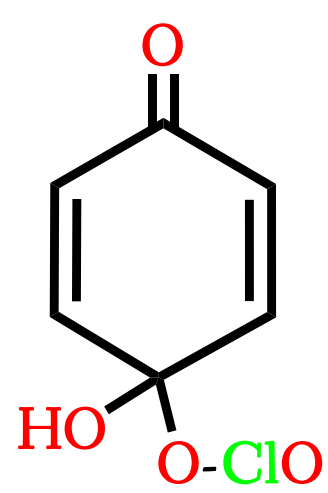

C -0.42908100 -0.62776100 -0.86688000
C -1.67835500 -1.02643800 -1.08274800
C -2.75084900 -0.78748800 -0.09141100
C -2.38518800 -0.03682100 1.12954600

C -1.13810700 0.36484700 1.35286200
C -0.01718900 0.09652500 0.38645500
H -3.18380000 0.17480500 1.83084100
H -0.85941400 0.91953000 2.24177600
O -3.88668800 -1.18506600 -0.27786200
O 0.84685100 -0.76163100 1.19661300
Cl 2.35379000 -1.15851600 0.47732800
O 2.24487100 -2.43600000 -0.37384600
H -1.95986700 -1.54500900 -1.99158700
H 0.35122900 -0.81238900 -1.59995100
O 0.61757600 1.29777300 0.11905100
H 1.00239600 1.29886900 -0.76243000

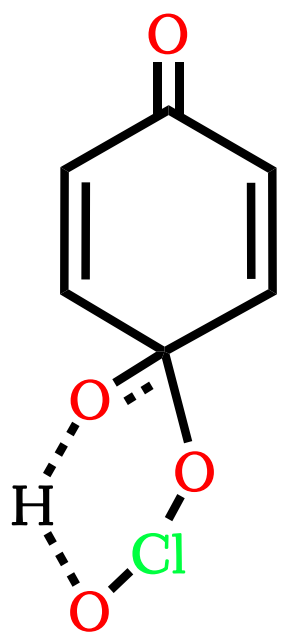

C -0.20704900 0.23411200 0.29776100
C 0.05548800 0.20832000 1.60461400
C 1.44865200 0.07687200 2.10595800
C 2.53315600 -0.02501000 1.10055600
C 2.26660600 -0.02940400 -0.20541400
C 0.88082500 0.11450800 -0.67281200
H -1.20752600 0.34082700 -0.10205200
H -0.72511400 0.29032000 2.35123100
H 3.54511800 -0.09836800 1.47971800
H 3.03781200 -0.11012200 -0.96108500
O 1.68517200 0.05327100 3.29669000
O 0.88155400 -2.02182200 -1.02159500
Cl 0.85788400 -2.16832600 -2.62114200
O 1.92677400 -1.09676300 -3.20472100
O 0.62495300 0.50254200 -1.86655500
H 1.31583700 0.09728600 -2.54384000

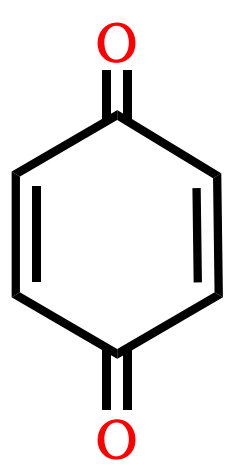

C -0.66689700 -1.26983600 -0.00003400
C 0.66689700 -1.26983600 0.00006500
C 1.43553800 0.00000000 0.00031600
C 0.66689700 1.26983600 0.00006900
C -0.66689800 1.26983600 -0.00003000
C -1.43553800 0.00000000 -0.00036500
H -1.24653500 -2.18598900 -0.00000800
H 1.24653500 -2.18598900 0.00008400
H 1.24653500 2.18598900 0.00009100
H -1.24653500 2.18598900 -0.00000100
O 2.65155800 0.00000000 -0.00017700
O -2.65155800 0.00000000 0.00024300

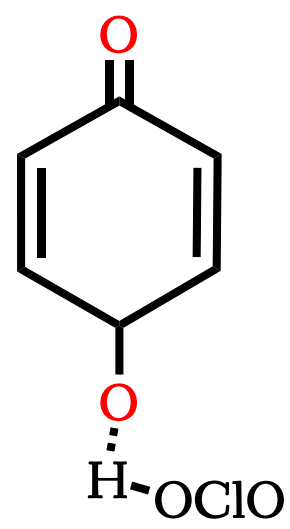

C -2.49929900 1.46291400 -0.73019500
C -1.17601900 0.90219600 -0.70986300
C -0.98787700 -0.32883700 0.09438600
C -2.10459700 -0.89126100 0.76024700
C -3.37840600 -0.30314000 0.67931700
C -3.57218400 0.86313200 -0.05668800
H -2.63861000 2.38170300 -1.28773900
H -1.95093300 -1.80206800 1.32667900
H -4.20695500 -0.75829000 1.20683500
O 0.15217500 -0.91854300 0.15223100
H 1.18900900 -0.31460200 -0.10277900
Cl 3.42623400 -0.75255100 0.45351100
O 3.84035700 -1.93104600 -0.47968100
O 2.25395900 0.18840800 -0.19022300
H -0.35583800 1.34013900 -1.23940100
O -4.71469700 1.38705500 -0.11783200

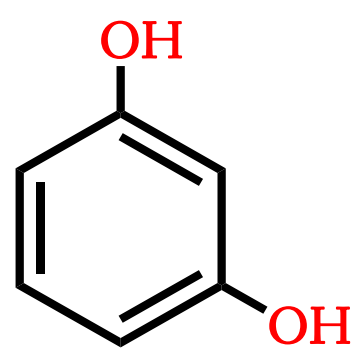

C -1.84408900 -1.28718800 0.01771600
C -2.35264500 0.00773000 -0.00707700
C -1.51475100 1.11321100 -0.00507800
C -0.13518000 0.91260700 0.02231200
C 0.39732700 -0.37161900 0.04829100
C -0.46375300 -1.46622300 0.04586100
H -3.42660100 0.15461300 -0.02920800
H -1.90979400 2.12137700 -0.02488600
O 0.65734900 2.01853300 0.02241900
H 1.58313400 1.76350700 0.03797800
H -2.50929600 -2.14387100 0.01509400
O 0.11139900 -2.69885500 0.07248200
H -0.56865200 -3.37706300 0.07139300
H 1.46940400 -0.53481800 0.07131300

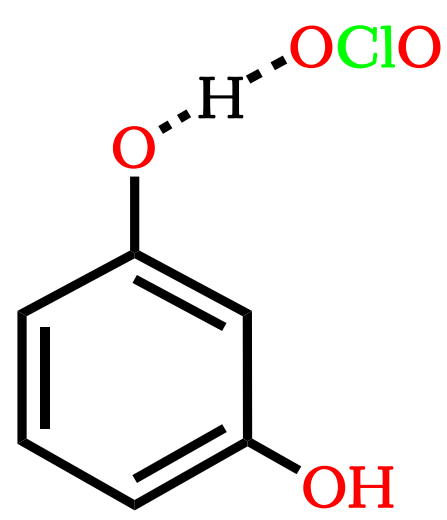

C -1.97978700 1.57913800 -0.26056100

C -1.01050900 0.80735600 -0.86281200
C -0.96883800 -0.58184800 -0.59989400
C -1.92298600 -1.18176100 0.27204900
C -2.88720900 -0.39284700 0.85101800
C -2.92542400 0.98197600 0.59879400
H -1.86009700 -2.24657700 0.45367900
H -3.62240700 -0.82869500 1.51519000
H -3.68795200 1.59510200 1.06623400
O -0.08452600 -1.36060100 -1.16847400
H 0.82725800 -0.87376400 -1.24809600
Cl 1.79611000 0.32318100 0.67595800
O 2.28920400 -0.70275200 1.72006800
O 2.02661100 -0.18401200 -0.80930600
O -1.99269800 2.89818100 -0.53024100
H -2.68225200 3.34753300 -0.03351300
H -0.29380100 1.25218100 -1.54128300

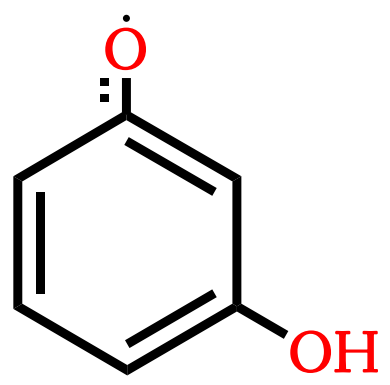

C -0.70835700 -1.41233700 0.08986000
C -0.04490300 -0.21507000 0.09795900
C -0.77694900 1.02839100 0.04144200
C -2.22831800 0.95964800 -0.02437100
C -2.86362700 -0.25595200 -0.02989300
C -2.12489500 -1.44455400 0.02658200
H -2.77515000 1.89373700 -0.06832500
H -3.94425600 -0.31205200 -0.07940500
H -2.63896300 -2.39974600 0.02163200
O 0.01592900 -2.55392300 0.14340000
H -0.55748800 -3.32453100 0.12506600
H 1.03666200 -0.17817500 0.14703400
O -0.19037400 2.13003900 0.04630800

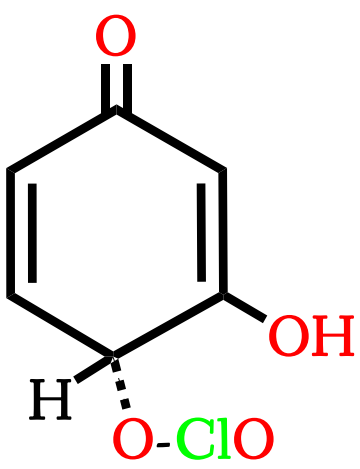

C 1.56886900 -1.36877300 -0.84434100
C 1.74364600 -0.05588400 -1.14948700
C 1.46537300 0.98318900 -0.14946300
C 1.02629800 0.54947800 1.19889300
C 0.84405100 -0.76472300 1.45704800
C 1.00113500 -1.70855400 0.41467400
H 0.88305700 1.32015700 1.94519700
H 0.50344200 -1.10952800 2.42406700
H 0.76633700 -2.74963600 0.61477700
O 1.61885500 2.16786100 -0.40480400
O -0.81507500 -0.43945100 -0.76384300
Cl -2.21682500 0.06430200 -0.26374000
O -2.16083000 0.49275900 1.22659000
O 1.83618900 -2.30825500 -1.77117500
H 1.67746200 -3.19241400 -1.42928700
H 2.09636800 0.25968800 -2.12347400

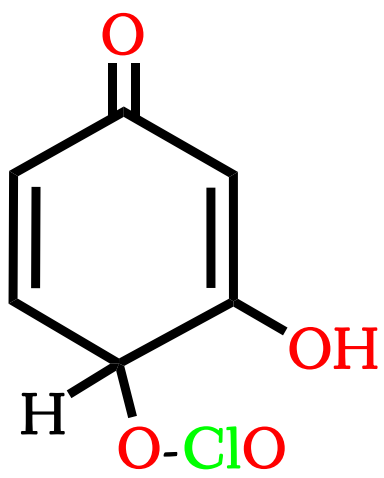

C -0.02522200 -0.27746100 0.43774100
C 1.23540800 0.18091900 0.37513200
C 1.51972600 1.59292700 0.13945800
C 0.36343700 2.50350200 -0.04010700
C -0.88763600 2.06064000 0.03334500
C -1.21927300 0.63162300 0.32683900
H 0.59043900 3.54348300 -0.24341900
H -1.73384700 2.72449500 -0.10748200
O 2.66522300 2.02099400 0.08456500
O -2.04794700 0.09585300 -0.72836300
Cl -3.72425600 0.12237500 -0.36651800
O -4.07022500 -0.96563000 0.65985000
H -1.76048100 0.56070500 1.27919800
H 2.08237900 -0.48413700 0.50356100
O -0.38122600 -1.54517000 0.64989900
H 0.39429600 -2.10998200 0.73369200

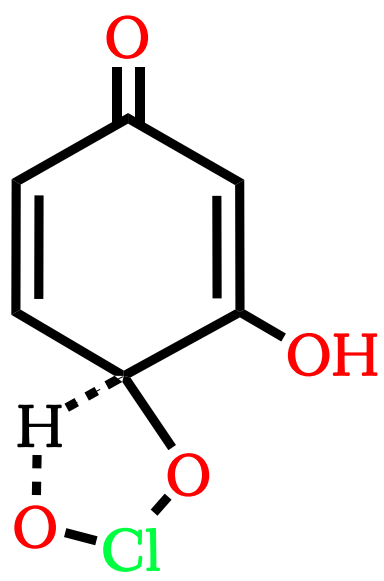

C -0.10692500 1.89014500 -0.37474100
C 1.17570900 2.26568500 -0.51582700
C 2.26897000 1.32048200 -0.31662700
C 1.91602200 -0.06486600 0.07336100
C 0.65025400 -0.44935700 0.21012900
C -0.49917800 0.47209400 -0.05295900
H 1.43858900 3.28350800 -0.78328400
H -1.05274100 0.10223300 -0.94004300
O -1.37388500 0.48092000 1.05788000
Cl -2.96943400 -0.24903000 0.65720100
O -3.07704900 -0.37592100 -0.87176600
O -1.17172000 2.67756600 -0.53292900
H -0.90639700 3.57717400 -0.75107500
H 2.74118200 -0.74458600 0.25212100
H 0.38770600 -1.45853300 0.50803000
O 3.44128800 1.64969200 -0.45617900

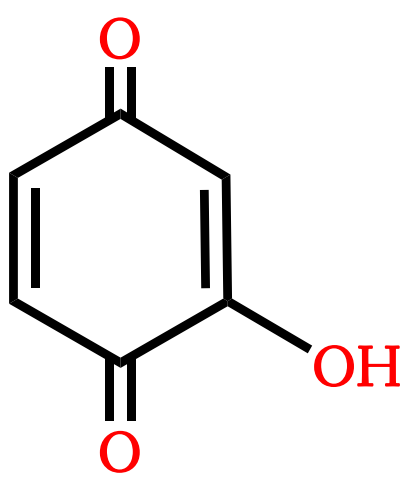

C 0.73968100 -1.08991800 0.09199400
C -0.15989300 -0.09118000 0.10776900
C 0.27748400 1.35062500 0.05288300
C 1.73140800 1.61065800 -0.02160100
C 2.61272300 0.61210800 -0.03762300
C 2.17415100 -0.81098000 0.01855200
H 0.44128000 -2.13179000 0.13316800
H 2.02640700 2.65257700 -0.06289300
H 3.68163500 0.78282700 -0.09373200
O -0.53514700 2.24725300 0.06866200
O 3.00287800 -1.70604800 0.00414800
O -1.48251200 -0.22613200 0.17109900
H -1.73066900 -1.15610300 0.20068800

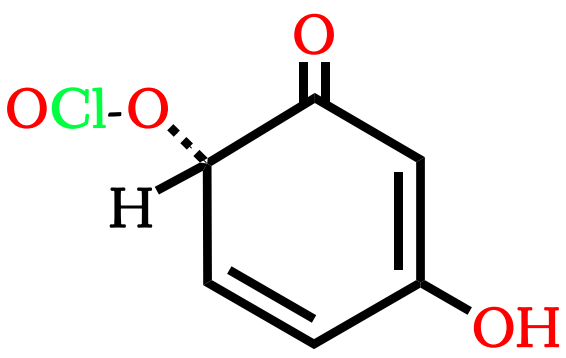

C 1.58575000 -1.20751900 -0.82859300
C 1.83161600 0.16827900 -0.98150200
C 1.43098600 1.11807500 -0.03618500
C 0.75930500 0.70682000 1.09271800
C 0.51934900 -0.72349700 1.32021700
C 0.91666700 -1.67158800 0.26640400
H 1.92408700 -1.88788200 -1.59907100
H 2.37118400 0.50389200 -1.86208400
H 0.74501200 -2.72261400 0.45166000
O -1.49397700 -1.52779900 -0.34965500
Cl -2.20199300 -0.54762900 0.60932600
O -1.53819200 0.84104800 0.45901100
O -0.07876700 -1.09905400 2.32457100
H 0.51180700 1.39481000 1.88925500
O 1.66473200 2.44420700 -0.18779000
H 2.13660000 2.61778400 -1.00650900

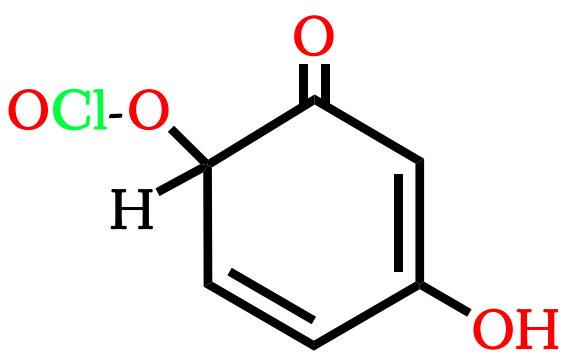

C 0.08952100 -0.30078200 -0.05326500
C 1.30100900 0.24911900 0.00718100
C 1.45568100 1.68626700 0.24187800
C 0.39743100 2.53622600 0.31099200
C -0.93793900 2.03716400 0.12814500
C -1.12404200 0.51751500 0.23265700
O -2.17554400 0.07433400 -0.62134600
Cl -3.74288700 0.39489700 0.01487800
O -4.31912900 -0.89867400 0.61238700
H -0.05300500 -1.35964700 -0.23065900
H -1.38709300 0.32595600 1.28551200
O -1.92356200 2.73489700 -0.06181100
H 2.19272200 -0.35305200 -0.12872100
O 2.68751100 2.18714200 0.35136500
H 3.35822400 1.50317500 0.26289400
H 0.55385400 3.60483700 0.38612300

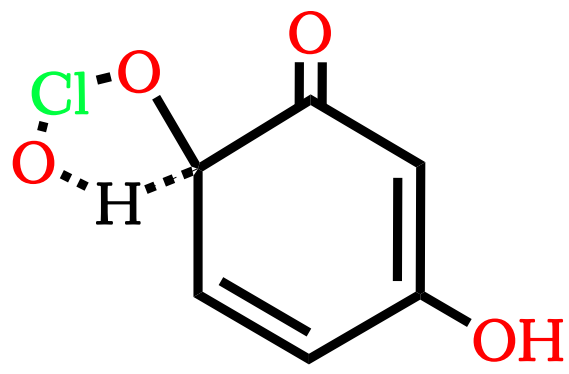

C -0.15608800 1.94297800 -0.23056800
C 1.09261100 2.29531200 -0.53017300
C 2.15803500 1.29814400 -0.52555600
C 1.95988300 0.00225000 -0.16669300
C 0.66055200 -0.46074200 0.25056400
C -0.51282600 0.51780500 0.04135400
H -0.96287400 2.66646700 -0.20900200
H 1.36249400 3.31768100 -0.76388400
H -0.98578600 0.11235800 -0.88277500
O -1.41267200 0.40352900 1.10640600
Cl -3.05753100 -0.07388200 0.52468100
O -2.89198000 -0.66110500 -0.88885000
O 0.44206300 -1.57363600 0.69745000
H 2.78528100 -0.69924100 -0.11414700
O 3.34466900 1.79566900 -0.87969500
H 4.02525100 1.11462100 -0.85520100

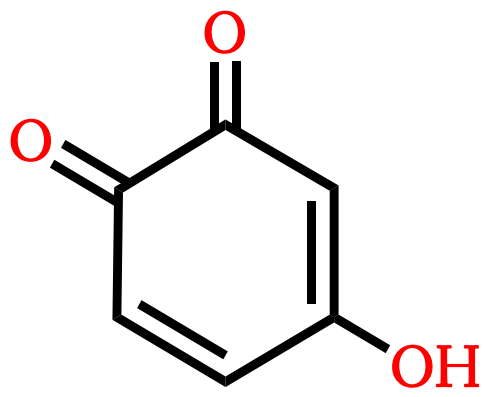

C -1.70099000 0.14548700 -0.09100800
C -0.23161400 0.17307800 -0.18490700
C 0.47963300 1.32457100 -0.16895600
C -0.19301600 2.59403000 -0.05405800
C -1.74967600 2.57642500 0.05265000
C -2.42012900 1.26502400 0.01945500
H -3.50079700 1.24976200 0.09012700
O 0.37278700 3.67088300 -0.03304200
O -2.35551100 3.61601000 0.15867500
H -2.19063000 -0.82295000 -0.11428000
O 0.41421500 -0.98915400 -0.29018900
H -0.19028600 -1.73753000 -0.28897500
H 1.55985900 1.30965500 -0.24165800

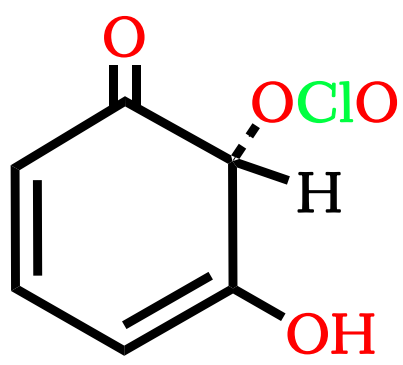

C 1.56974600 -1.22214400 -0.83639200
C 1.83848100 0.14120300 -0.98218800
C 1.45733400 1.09892800 -0.02573200
C 0.78523600 0.72252400 1.10013400
C 0.51699400 -0.70539800 1.33109500
C 0.89042300 -1.66096800 0.27758000
H 2.37789400 0.45840600 -1.86808900
H 0.71930600 -2.71041400 0.48056700
O -1.36998300 -1.49307900 -0.39235400
Cl -2.20723500 -0.62880300 0.58380400

O -1.68694700 0.82082500 0.51842900
O -0.08071700 -1.09015000 2.33140200
H 0.51838500 1.41289600 1.88776600
O 1.98352000 -2.03882300 -1.83566900
H 1.74562300 -2.95085200 -1.64889700
H 1.69830500 2.13904800 -0.20264200

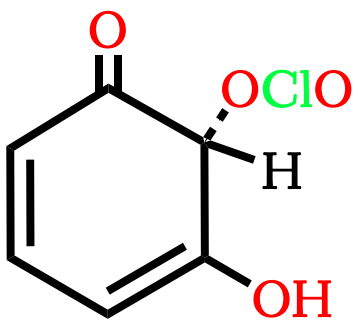

C -0.13300600 1.92743200 -0.36258300
C 1.28201400 2.23847700 -0.51618800
C 2.22285000 1.31188800 -0.25356500
C 1.92118600 -0.04841400 0.16347200
C 0.65500500 -0.45719700 0.26135800
C -0.48908900 0.44384900 -0.12056500
H 1.53874500 3.25833900 -0.77485000
H -0.87848400 0.10352500 -1.09257100
O -1.51951900 0.40763500 0.86166700
Cl -2.77190100 -0.71993000 0.52918400
O -3.77490900 -0.12139500 -0.46732900
H 3.26976600 1.58401200 -0.33214600
O 0.29602500 -1.69175500 0.71619300
H 0.23838800 -2.32104100 -0.00965500
O -1.02115900 2.74981300 -0.44141900
H 2.73111900 -0.72687100 0.40025600

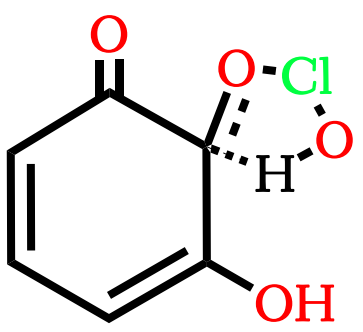

C -0.13558800 1.93392300 -0.23722100
C 1.12521900 2.29593800 -0.53234200
C 2.17683900 1.30888100 -0.50066500
C 2.00019100 0.01153800 -0.16235600
C 0.69546500 -0.47968900 0.23122400
C -0.48783900 0.49456800 0.02066300
H 1.36633700 3.32233800 -0.78155800
H -0.94680800 0.10395100 -0.91197700
O -1.38596900 0.34190700 1.08551400
Cl -3.05873200 0.14106700 0.51568500
O -3.01376600 -0.61293800 -0.82433500
O 0.47636400 -1.59423300 0.66359600
H 2.83078000 -0.68117200 -0.10840600
H 3.17646100 1.65078000 -0.74826200
O -1.20500200 2.73466800 -0.19976200
H -0.96122300 3.64464900 -0.39865200

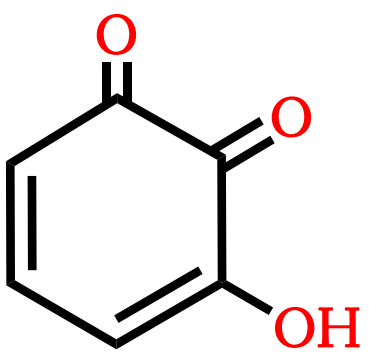

C -1.67351700 0.12721100 -0.14012900
C -0.21988000 0.18095700 -0.19076500
C 0.49856200 1.31759700 -0.14767600
C -0.18585600 2.59655900 -0.04304000
C -1.74901000 2.57155600 0.02520000
C -2.41827600 1.24350600 -0.04030400
O 0.36456500 3.67497600 -0.00526700
O -2.35963400 3.60415000 0.12551600
H -2.15001100 -0.84550600 -0.18846700
H 1.58041600 1.32063200 -0.19069900
H 0.29475500 -0.77015200 -0.27045700
O -3.75469900 1.31137700 0.01263000
H -4.14070000 0.43061200 -0.02955400

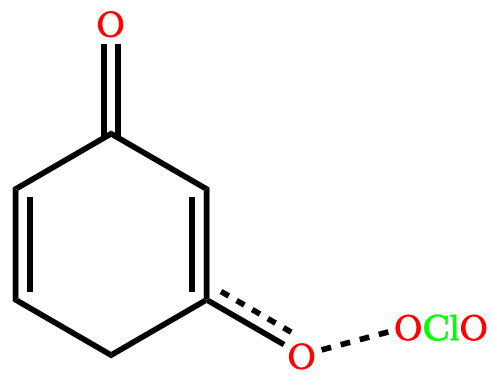

C -2.19746400 1.51209100 -0.52486700
C -1.08785600 0.64323300 -0.51318800
C -1.05908400 -0.67418400 0.09870900
C -2.22164700 -1.11374900 0.67649500
C -3.40903700 -0.26917500 0.68882600
C -3.34768500 1.09469500 0.05233300
H -2.10936100 2.47978100 -0.99925500
H -2.29149900 -2.08901400 1.14066800
H -4.25119000 1.69165200 0.08452500
O 0.03088100 -1.37562600 0.06144800
H 0.92221200 -0.85616400 -0.42941500
Cl 3.02237900 0.19908300 0.05093600
O 4.06059800 -0.95967300 0.27480200
O 1.86465300 -0.25639100 -1.00918600
H -0.16810800 0.96878200 -0.99521800
O -4.45670500 -0.61713800 1.19540700

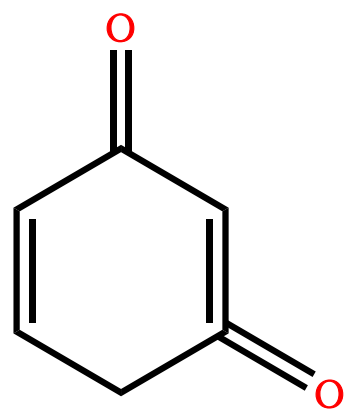

C -2.16080500 1.52395500 -0.54105900
C -1.07247400 0.67976300 -0.53227600
C -1.07592700 -0.69030000 0.09956000
C -2.26534400 -1.12823100 0.69666600
C -3.40581500 -0.31382000 0.70944200
C -3.30450300 1.04244600 0.05584100
H -2.12295800 2.50212000 -1.00098900
H -2.30183000 -2.10527500 1.16126800
H -4.21354800 1.63643200 0.08401500
O 0.00485900 -1.29931900 0.02250900
H -0.13381500 0.97346200 -0.99330300
O -4.51659500 -0.56988400 1.20420800
